# Utility-Driven End-to-End Network Slicing for Diverse IoT Users in MEC: A Multi-Agent Deep Reinforcement Learning Approach

**DOI:** 10.3390/s24175558

**Published:** 2024-08-28

**Authors:** Muhammad Asim Ejaz, Guowei Wu, Adeel Ahmed, Saman Iftikhar, Shaikhan Bawazeer

**Affiliations:** 1 School of Software Technology, Dalian University of Technology, Dalian 116024, China; 2 Department of Computer Science, Faculty of Computing, The Islamia University of Bahawalpur, Bahawalpur 63100, Pakistan; adeelmcs@gmail.com; 3 Faculty of Computer Studies, Arab Open University, Riyadh 84901, Saudi Arabia; s.iftikhar@arabou.edu.sa (S.I.); shaikhan@arabou.edu.sa (S.B.)

**Keywords:** Internet of Things (IoT), mobile edge computing (MEC), end-to-end network slicing, multi-agent, deep reinforcement learning (DRL), utility optimization

## Abstract

Mobile Edge Computing (MEC) is crucial for reducing latency by bringing computational resources closer to the network edge, thereby enhancing the quality of services (QoS). However, the broad deployment of cloudlets poses challenges in efficient network slicing, particularly when traffic distribution is uneven. Therefore, these challenges include managing diverse resource requirements across widely distributed cloudlets, minimizing resource conflicts and delays, and maintaining service quality amid fluctuating request rates. Addressing this requires intelligent strategies to predict request types (common or urgent), assess resource needs, and allocate resources efficiently. Emerging technologies like edge computing and 5G with network slicing can handle delay-sensitive IoT requests rapidly, but a robust mechanism for real-time resource and utility optimization remains necessary. To address these challenges, we designed an end-to-end network slicing approach that predicts common and urgent user requests through T distribution. We formulated our problem as a multi-agent Markov decision process (MDP) and introduced a multi-agent soft actor–critic (MAgSAC) algorithm. This algorithm prevents the wastage of scarce resources by intelligently activating and deactivating virtual network function (VNF) instances, thereby balancing the allocation process. Our approach aims to optimize overall utility, balancing trade-offs between revenue, energy consumption costs, and latency. We evaluated our method, MAgSAC, through simulations, comparing it with the following six benchmark schemes: MAA3C, SACT, DDPG, S2Vec, Random, and Greedy. The results demonstrate that our approach, MAgSAC, optimizes utility by 30%, minimizes energy consumption costs by 12.4%, and reduces execution time by 21.7% compared to the closest related multi-agent approach named MAA3C.

## 1. Introduction

The rapid growth of the Internet of Things (IoT) and mobile Internet has significantly increased user data traffic on edge networks. Cloud computing provides on-demand services such as data processing and storage to users. However, due to limited resource availability in cloud servers, this can lead to excessive bandwidth utilization, as well as data transfer and latency issues [1,2]. For example, in an emergency situation where users require rapid real-time responses, additional latency and obtaining resources from the cloud server could result in a major disaster. The current scenario encourages the distribution of computational resources from the cloud server to the edge of the network [3]. Advancements in machine learning (ML) techniques and artificial intelligence (AI) have enabled the rapid processing and training of immense amounts of data. This progress not only allows for adaptation to dynamic environments but also leads to a considerable reduction in latency and improvement in computational performance [4,5]. Mobile edge computing (MEC) intelligence, the combination of MEC with ML and AI, will undoubtedly be a significant booster for the potential advancement of networks. However, these developments may pose major challenges when resources are scarce in MEC cloudlets and users demand an urgent response with quality of services (QoS) [6,7,8,9].

In the MEC paradigm, network service providers (SPs) lease resources in cloudlets deployed at various locations near IoT users in a large area. Users utilize these resources, and SPs charge for their usage to maximize profit [10]. Therefore, in dense areas such as shopping malls and city centers—and in contrast to rural areas—where a high concentration of IoT users surrounds a single cloudlet, efficiently providing services becomes challenging due to the limited availability of cloudlet resources. Effectively utilizing MEC resources and balancing utilization across cloudlets is a complex task, particularly when user requirements demand minimal latency and urgent responses [11]. When IoT users send network slicing requests to the nearest cloudlet, these requests can have varying requirements. Some tasks might be flexible with processing times, while others may demand the fastest possible computing power to prevent significant user losses.

The limited computing capacity of cloudlets, while enabling the provision of diverse services to IoT users, presents a significant challenge in optimizing network slicing resources within our approach. This capacity is represented by the availability of Virtual Network Function (VNF) instances in activated or deactivated states within cloudlets. Chaining these VNFs allows for the creation of network slices that meet the demands of IoT users [12]. However, effectively fulfilling both common and urgent request requirements from a high volume of IoT users becomes difficult due to these resource constraints. In scenarios with a large number of IoT users, available cloudlet resources may be constrained, leading to challenges in handling numerous simultaneous requests. Specifically, when approximately 1000 IoT requests arrive at the same time, resource limitations can result in delays of up to 30% for urgent requests and a reduction of 20% in overall efficiency due to inadequate distribution [13].

ML techniques such as deep reinforcement learning (DRL) and reinforcement learning (RL) have been employed by scholars to address the issues mentioned earlier. These techniques have demonstrated their significant impact in resolving real-world optimization challenges, including resource allocation, computational outsourcing, adapting to environmental changes, and maintaining QoS. This efficiency is particularly notable in the environment of network slicing, including MEC and IoT [14].

Developing an effective resource optimization technique is critical for MEC. This technique should optimize scarce cloudlet resources, minimizing latency and cost while maximizing SP utility. Various prior works have investigated resource allocation in MEC, focusing on historical traces for joint request placement and resource allocation [15], adaptive VNF deployment and migration of network slices [16,17], maximization of utility by accepting the maximum number of network slicing requests [18], and maximization of SP revenue by providing on-demand network slicing [19]. However, these approaches often lack the necessary adaptability to rapidly changing and diverse environments, particularly when IoT users require urgent computing in the MEC network. By implementing real-time resource allocation mechanisms, MEC can ensure that urgent IoT user requests are fulfilled promptly, potentially mitigating disaster risks.

Our primary objective is to develop a resource optimization technique for MEC that can dynamically allocate resources to meet the demands of both urgent and common IoT user requests. This approach aims to achieve a balance of cloudlet resource utilization, ensuring timely completion of common tasks while prioritizing urgent requests with minimal latency. All resource allocation decisions are made while adhering to established QoS requirements. However, despite considerable improvements in achieving time-sensitive operations, potential challenges remain. These include limited resources in cloudlets, congestion in links due to restricted available bandwidth, and the lack of precise prediction of request requirements. Centralizing all requests, both common and urgent, on the primary cloudlet can provide low-latency responses for users. However, this approach introduces a single point of failure and a resource bottleneck. Even if additional VNF instances are activated, the limited capacity of the primary cloudlet may prove insufficient for sustained high volumes of diverse requests [20]. A key challenge lies in dynamically deciding to process requests locally or not, considering system constraints. DRL, in conjunction with network slicing within MEC, offers a promising solution. In a multi-time-slot scenario where IoT user requests arrive randomly, we investigate the use of DRL to address the challenge of end-to-end network slicing in MEC for network resource optimization [21,22]. Traditional DRL often utilizes centralized or decentralized learning with a single agent. However, this approach presents limitations for network slicing in MEC due to the following two key factors: a vast number of geographically dispersed cloudlets and a large, continuous action space [23].

In contrast to these studies, in our end-to-end network slicing utility optimization problem, we propose a multi-agent DRL approach that intelligently handles a large, continuous action space and accommodates the diverse needs of each user request. By avoiding premature convergence, we train the model to optimize system utility, maximize rewards, reduce energy consumption costs, mitigate execution times for both common and urgent user requests, balance the utilization of cloudlet resources, and achieve higher accuracy compared to existing methods. These aspects have not been well investigated in the current network slicing problem.

To be more precise, our approach evaluates the following crucial questions:

- How to prevent assigning all common and urgent requests to the home cloudlet to avoid overloading a single cloudlet location;
- How to decide which request to assign to the adjacent cloudlet;
- When it is necessary to activate a VNF instance and when to deactivate it;
- How to ensure that common requests are assigned within their deadlines, even when urgent requests are also arriving;
- How to manage available resources in the cloudlet if a user’s demand changes in real time.

Therefore, our work presents significant contributions to the field of network slicing in MEC, particularly regarding the efficient handling of both common and urgent IoT user requests with minimal latency. To address the previously identified challenges, this research proposes a multi-agent-based approach with the following key findings:

- We design a network slicing-based MEC system using multi-agent soft actor–critic (MAgSAC), where cloudlets are placed at various locations near IoT users to provide end-to-end resource allocation services. These cloudlets have computing capabilities in the form of VNF instances, which can be activated or deactivated as needed. This setup accommodates both common and urgent IoT user requests while balancing resource allocation across cloudlets, ultimately ensuring QoS.
- We propose an extensive optimization problem model that aims to optimize the overall utility of the MEC network. This is achieved through intelligent network slice utilization, which involves a trade-off between revenue, energy consumption cost, and overall execution time. By transforming this complex optimization problem into a DRL problem, we describe it as a Markov Decision Process (MDP) and approach it as a multi-agent DRL problem.
- We devise a multi-agent DRL-based MAgSAC algorithm, which intelligently provides resources for both common and urgent requests through prediction by activating and deactivating VNF instances in home cloudlets, as well as adjacent cloudlets. It minimizes energy consumption costs by reconsidering idle or remaining capacity before deactivating VNF instances, thereby maximizing overall utility and minimizing latency. This scheme efficiently facilitates user needs and prevents cloudlets from creating imbalanced network slicing during resource allocation. Our approach aims to intelligently handle the optimization challenges mentioned earlier.
- We conduct extensive simulations to compare our MAgSAC approach with benchmark methods, including MAA3C, SACT, DDPG, S2Vec, Random, and Greedy. The results indicate that our MAgSAC scheme achieves the highest utility with the lowest execution time, as well as minimum delay and energy consumption cost compared to the other approaches.

## 2. Related Work

Network slicing has recently attracted significant attention from researchers due to the challenges of efficient resource allocation and latency-aware optimization in MEC networks [24,25,26,27,28]. In network communication, ML approaches are rapidly developing as an emerging trend to improve QoS and solve optimization problems such as resource allocation in MEC [29] and real-time service provisioning for both common IoT users and those requiring urgent services [30]. State-of-the-art research focuses on ML approaches for resource optimization in MEC, highlighting this area as a key focus of existing studies.

For example, to balance edge network resources and user demand in bursty traffic, a multi-agent-based two-fold algorithm for resource allocation and request redirection (RA-RR) was introduced in [31]. This approach matches and coordinates slice resource demand using Lyapunov optimization theory to predict changes in demand and efficiently make coupled decisions. The authors of [32] optimized the problem of resource management, considering how previous actions impact the future long-term reward of MEC servers regarding computational latency and energy utilization. To solve this optimization problem, they introduced a multi-agent Deep Deterministic Policy Gradient (DDPG)-based strategy for resource allocation, which also reduces the action space. The authors of [33] proposed a cooperative multi-agent DRL (Coo-MADRL) method to tackle challenges in multi-cloud and multi-edge environments, such as network topology and bandwidth constraints. This method maximizes server and link resources while minimizing task latency, using centralized training and decentralized implementation to optimize rewards. The authors of [34] introduced a single- and multi-agent DRL-based one-shot agent scheme that autonomously manages network slice requests. The one-shot agent decides where to place these requests on the physical infrastructure to maximize the reward and the total number of accepted requests, thereby improving QoS.

A joint optimization model integrating resource constraints was proposed to address the joint optimization problem of service migration and resource allocation (SMRA). The authors of [35] introduced a DRL-based algorithm for SMRA, where Long Short-Term Memory (LSTM) predicts mobile user behavior and identifies an optimal resource allocation scheme, while a Parameterized Deep Q Network (P-DQN) resolves the migration policy to maintain service continuity. The authors of [36] addressed the issue of completing tasks before their deadlines while minimizing energy consumption and cost. They proposed an end-to-end DRL scheme to handle the large action space by maximizing rewards and offloading tasks to the best server, thereby mitigating computational costs. The authors of [37] used an intelligent natural actor–critic DRL to minimize energy costs and latency while maximizing available processing capacity (APC), thereby improving system performance and rewards. The authors of [38] proposed a DRL-based dueling DQN scheme to handle the resource allocation and energy cost minimization problem. This scheme addresses the issue in multi-tenant networks where lower-priority tenants dynamically change their behavior to that of high-priority tenants, potentially leading to increased energy consumption costs.

The authors of [39] addressed the issues of task delay and extra energy consumption regarding the joint offloading and resource allocation (JORA) problem in MEC networks, aiming to maintain the Quality of Experience (QoE) for end users. They introduced the Lyapunov optimization approach to maximize long-term QoE and an energy deficit queue to guide real-time, online solutions for the energy consumption problem. By addressing the issues of the Age of Information (AoI), energy consumption, and convergence, the authors of [40] focused on minimizing the AoI in a wirelessly powered IoT environment. The challenge of optimizing transmission selection, channel selection, transmission duration, and transmit power was demonstrated to be NP-hard. The proposed strategy, a distributed multi-node resource allocation method called distributed multi-node resource allocation (DDMRA), combines the DDPG approach with the discrete action selection technique from the DQN.The research reported in [41] focused on maximizing QoE by correlating it with QoS through joint task and resource allocation. A multi-agent DRL-based model for Distributed Joint Task and Computing Resource Allocation (DJTCRA) dynamically allocates resources to user tasks, partially observing real-time states in edge computing to enhance QoE.

To solve the network slicing optimization problem of computing, communication, and cache (3C), along with QoS in MEC, the authors of [42] maximized utility using a DRL-based twin-actor DDPG algorithm. This algorithm intelligently adapts to changes in the environment and effectively allocates resources. In [43], the authors aimed to maximize the expected utility based on the reservation of slices by introducing a two-time-scale scheme. In the long time scale, tenants decide whether to activate the slice or not, and in the short time scale, they reconfigure the active slice by adapting to user demand. The Frank–Wolfe algorithm was proposed to solve the long-time-scale convex approximation problem, while Least Absolute Shrinkage and Selection Operator (LASSO) regularization is used for short-time-scale slice reconfiguration.

To address resource utilization for VNF sharing and new-instance creation, the authors pf [44] introduced an integer linear programming (ILP)-based core network slicing (OCNS) model. They also proposed a heuristic backtracking algorithm for network slicing (HBA-NS) that allocates resources sequentially while considering service-level agreements (SLA). Additionally, a deep learning (DL)-based Convolutional Neural Network (CNN)+LSTM model predicts requests using historical data to adjust resources for future time slots. The study reported in [45] highlights network slicing as a key technology for next-generation networks, efficiently utilizing resources to increase the profit of SPs. The SLA-NS framework optimizes SP profit through network slice pricing, demand forecasting, and resource allocation. By employing a two-layer game model and an LSTM predictor, SLA-NS effectively decouples pricing and allocation to enhance efficiency. The authors of [46] proposed an online DRL-based model-free policy gradient strategy to address the problem of slice tenants (STs) requesting and selecting resources from infrastructure providers based on predefined configurations. A two-stage reward function (TRF) was designed to optimize the objective and minimize the cost for slice brokers (SBs).

To improve network slice mobility (NSM) through future predictions, the authors of [47] proposed a federated DRL (FDRL) mechanism. This approach improves system scalability and flexibility and boosts long-term profit using FDRL predictions and double deep Q learning (DDQN) for decision making. The authors of [48], introduced a probabilistic forecasting technique for the prediction of network slicing demand, building upon prior deterministic techniques. A DeepAR-based slice admission control mechanism was proposed for making sequential decisions in Software-Defined Networks (SDNs), supported by the SDN controller. Additionally, a closed-loop parameter-updating method was developed to enhance the admission control process. The efficacy of these methodologies was demonstrated using actual traffic data from real-world scenarios. The authors of [49] addressed power allocation and request acceptance by considering resource limitations, user priorities, system stability, and long-term performance. They introduced the following two approaches: prediction-aided weight DRL (PW-DRL) for online power allocation and request acceptance and trust region policy optimization (TRPO) for adaptation to environmental changes.

Compared to the aforementioned resource optimization studies, our proposed DRL-based MAgSAC end-to-end network slicing approach considers both common and urgent requests of IoT users on MEC networks. It aims to optimize utility, mitigate energy consumption costs, and maximize revenue when resources on the cloudlets are limited. Additionally, our approach balances resource consumption with minimal time in the cloudlets. In terms of previous extended DRL approaches, MAgSAC represents a way of integrating a multi-agent framework with an extensive reward function and a modern MDP model. This integration effectively manages the fluctuating nature of IoT traffic and resource limitations, resulting in higher efficiency in handling VNF instances and fair resource allocation among cloudlets.

## 3. Motivation

Our main goal is to deliver dynamic and flexible services to IoT users, optimizing utility by balancing revenue, energy costs, latency, and QoS. Users select services for specific periods, while service providers (SPs) allocate resources based on available capacity and QoS efficiency. We use various examples to substantiate this approach. IoT users have diverse needs; some require uninterrupted connectivity, while others need a flexible QoS. For instance, soil moisture sensors in agriculture monitor humidity at intervals, while autonomous vehicles need constant connectivity for real-time traffic updates and safety. In emergencies, such as fire alarms, immediate responses are essential.

These examples demonstrate that IoT requirements vary by situation. Unknown user demands and resource availability can severely impact QoS. Efficient resource use requires the identification of user needs and allocation resources accordingly. The reservation of resources can lead to either under- or over-utilization depending on whether IoT users make regular or periodic requests. A lack of requests can waste resources and increase costs, while simultaneous requests from multiple users can cause network congestion.

Congestion varies by time and location, with some areas experiencing higher demand at different times. SPs offer services based on their capacity and coverage, but some may struggle to meet specific service levels. For example, urgent requests with strict delay requirements may conflict with common requests needing flexible delays, leading to increased latency, higher costs, reduced profit, and degraded QoS without a proper strategy. Assigning all urgent requests to the primary cloudlet can reduce latency but may create an imbalance in resource allocation, overburdening one site. To the best of the authors’ knowledge, existing research does not adequately address the challenge of balancing urgent and normal requests across cloudlets while optimizing overall utility and QoS.

## 4. System Model

**Figure 1:**
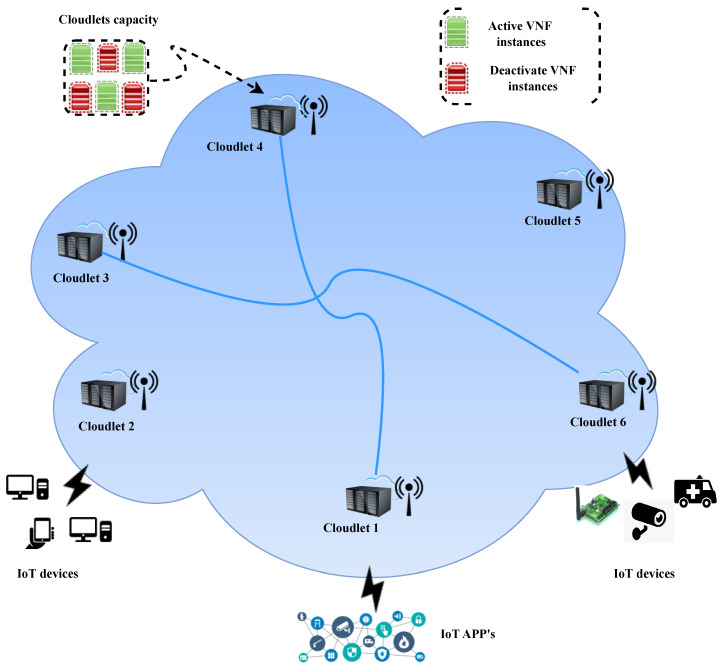
MEC-based system model.

Consider a mobile edge computing network represented by $G=\left(Z\cup V,E\right)$ in which V denotes a set of access points (APs), i.e., $V=\left\{v_{1},v_{2},v_{3},\ldots ,v_{n}\right\}$ and E describes the set of edges in the network, i.e., $E=\left\{e_{1},e_{2},e_{3},\ldots ,e_{n}\right\}$. In this edge computing network, resource-limited cloudlets, denoted by $Z=\left\{z_{1},z_{2},z_{3},\ldots ,z_{n}\right\}$, are placed at various locations close to users and are interconnected to perform end-to-end network slicing. Each cloudlet ($z_{j}\in Z$) has virtualized computing resources available as VNF instances to implement the different requested services in G. Let $F_{l}\in \mathrm{VNF}$ and l={1,2,3,….,|F|} represent the set of various VNF instances at each $z_{j}$, indicating the capacity ($z_{j}^{cap}$) of Z in the form of activated ($f_{l}^{⊕}$) and deactivated ($f_{l}^{⊘}$) VNF instances. We assume that in each cloudlet ($z_{j}$), a VNF instance ($f_{l}$) can be assigned to one IoT user request at a time, but the nature and demand of the user request need to be estimated in advance. Let $p(f_{l})$ denote the computing resource requirement of the VNF instance ($f_{l}$). Additionally, we assume that the monitoring time period (*T*) is divided into equal time slots denoted by *t* and indexed by 1≤t≤T. A limited amount of bandwidth (*B*) is available for each cloudlet to transmit and receive data. Let $D_{e}$ and $B_{e}$ be the delay and bandwidth resource capacity of each edge (E) of the cloudlet ($z_{j}$), respectively. The cloudlets ($z_{j}$) are interconnected with a set of links (E(i,j)). In G, via v∈V, the cloudlets Z receive the network slice requests ($r_{i}^{s}\in R^{s}$), with delay requirements sent by users from various IoT devices to the nearest $z_{j}$ in that area. Figure 1 illustrates the MEC network, where APs with interconnected cloudlets have the capacity to provide end-to-end network slicing services to IoT users in the form of activated and deactivated VNF instances. The terminology employed in this paper is defined in Table 1.

### 4.1. IoT Users Requests

In MEC networks, cloudlets (Z) are located at different locations, and the number of incoming IoT user requests ($r_{i}^{s}\in R^{s}$) varies from time to time and area to area. For example, during peak hours in a shopping mall, the number of incoming requests from IoT users can be significantly higher than in a residential area during the same time. Each user request ($r_{i}^{s}$) is received by the nearest cloudlets through *v*. Cloudlet $z_{k}$, which is near a shopping mall, airport, or train station, is much busier than cloudlets in rural areas. Due to the limited availability of computing resources in each cloudlet, it is hard to handle all the requests ($r_{i}^{s}$). Cloudlet $z_{j}$ sends overloaded requests to the adjacent cloudlet ($z_{k,i}\subseteq z_{k,j}$) in the case of the unavailability of the required resources to avoid imbalance in data overhead and latency issues.

By considering the continuous agility of IoT users moving between different cloudlets, we assume that incoming requests for required resources arrive randomly from various IoT users with an arrival rate of λ. Therefore, the arrival rate of user requests is denoted by

(1) $$\lambda =\frac{Z[r_{i}^{s}.p\left(f_{l}\right)]}{t},$$

where $r_{i}^{s}$ represents the demand of the user for computing resource ($p(f_{l})$) from the set of cloudlets (Z) at time slot *t*.

We denote the flow of requests from one cloudlet to another as F(i,j). To ensure that all incoming requests are accommodated, we ensure that the flow (*F*) of incoming requests does not exceed the edge-link capacity, i.e., $F\left(i,j\right)\leq E^{cap}$. We assume that if the sum of incoming requests equals the sum of outgoing requests, denoted as $\sum T_{in}=\sum T_{out}$, there is no rejection of requests and no delay in request processing.

It is important to remember that $\varrho_{j,l}^{t}$ represents the total number of unfinished requests for service ($f_{l}$) during the resource allocation phase of time slot *t*. Therefore, we denote the unfinished requests as follows:

(2) $$\varrho_{j,l}^{t}=\sum_{r_{i}^{s}\in f_{l}\cap R^{s,t}}\rho_{i,j}^{t},\;\forall i,j,l,t,$$

where $\rho_{i,j}^{t}$ indicates that the request ($r_{i}^{s}$) is currently being processed by cloudlet $z_{j}$ in time slot *t*.

### 4.2. Common and Urgent Requests

IoT users send heterogeneous kinds of requests to fulfill their specific requirements. Based on the demand, we divide these requests into two categories.

#### 4.2.1. Common Requests

IoT users’ requests that have specific resource demands but not an urgent deadline are considered common requests ($r_{i}^{s}$). Moreover, it is accurate to characterize these requests as less critical. A few examples of common requests include real-time video conferences, room temperature monitoring, and virtual reality applications.

#### 4.2.2. Urgent Requests

IoT users’ requests that arrive earlier than the regular period and demand strict and rapid assignment are classified as urgent requests ($r_{urg}^{s}$). Missing the deadline for these requests may lead to significant disasters and system failures. Urgent requests typically involve delay-sensitive requirements and an immediate response from the SP to mitigate further substantial losses. For instance, in natural disaster situations, an ambulance requires a prompt response to identify a shorter and less congested traffic route or a fire alarm sensor continuously analyzes data to prevent major damage. These types of IoT requests fall under urgent requests. MEC resources are deployed in such circumstances to process the sensed information and make swift decisions based on the collected data. In other words, the faster the response from the MEC network, the better the mitigation of major losses. To further distinguish between common and urgent requests, we introduce the following variable:

(3) $$r_{urg}^{s}=\left\{\begin{array}{cc} 1, & \mathrm{urgent}\;\mathrm{requests}\\ 0, & \mathrm{otherwise}. \end{array}\right.$$

In the MEC network, the system differentiates between common and urgent requests based on several factors, including IoT users’ application type, request requirements/characteristics, and priority level. Through the analysis of information collected from sensors, input sources, and historical records, relevant characteristics are identified and utilized to ascertain the nature of the requests, their sensitivity, the location of request origination, and environmental conditions. To distinguish between regular requests and urgent requests in human body monitoring and autonomous vehicle systems, T-distribution modeling [50] and anomaly detection approaches [51] are utilized to ensure effective and timely responses. In emergencies, these approaches respond effectively, allocate resources promptly according to the requirements, and ensure safety.

By integrating the previously outlined approaches of autonomous vehicles, we formulate an estimation range encompassing both upper and lower bounds based on the historical record of characteristics, required reaction time, location, and environmental factors [52]. The T distribution, denoted by $SD_{i}$, represents the sample characteristic associated with the response time for a normal sample size of *n*, where n>0. The sample mean value ($\bar{SD}$) is calculated as follows:

(4) $$\bar{SD}=\frac{\sum_{i=1}^{n}SD_{i}}{n-1}.$$

Therefore, the sample characteristics of the standard deviation can be defined by Δ as follows:

(5) $$\Delta =\sqrt{\frac{1}{n-1}\sum_{i=1}^{max}SD_{i}-\bar{SD}}.$$

Therefore, the upper and lower bound can be described as follows:

(6) $$U^{up}=\bar{SD}-\sqrt{\frac{1}{n-1}}.\Delta .\eta ,$$

(7) $$U^{lo}=\bar{SD}+\sqrt{\frac{1}{n-1}}.\Delta .\eta ,$$

where η denotes the average T-distribution coefficient for a sample size of *n*. The appropriate range for each monitored parameter of autonomous vehicles should fall within the ranges of Equations (6) and (7), which indicate the appropriate response time for the vehicle. At a given time slot (*t*), $U^{t}$ depicts the parameter of the recorded value of autonomous vehicles. Therefore, urgent requests at time slot *t* can be calculated as follows:

(8) $$r_{urg}^{s}=\left[\frac{{\left(U^{up}-U^{t}\right)}^{2}-{\left(U^{lo}-U^{t}\right)}^{2}}{{\left(U^{up}-U^{lo}\right)}^{2}}\right].$$

Higher values of $r_{urg}^{s}$ indicate that the corresponding requests demand an urgent response, and lower values indicate that the request can be dealt with normally if $r_{urg}^{s}=0$. In some situations, the value of the $r_{urg}^{s}$ may be theoretically greater ($r_{urg}^{s}>1$). To ensure that the value remains uniform throughout, we constrain the maximum value by adjusting the urgent-request demand level to $r_{urg}^{s}=1$, which is the highest possible level of urgent requests. To ensure uniformity in value retention, we constrain the maximum value by adjusting the urgent-request demand level to $r_{urg}^{s}=1$, representing the highest possible level of urgent requests for demanding resources. For clarity regarding response time for urgent and common requests, we establish a reference range with an upper bound of $U^{up}=5.5$ ms and a lower bound of $U^{up}=3.5$ ms [53]. A required response time of 3 ms indicates an urgent request, which must be rapidly assigned to the relevant cloudlet. Computing urgent requests according to user requirements requires a specific mechanism, clear end-to-end communication, and an efficient resource allocation approach to QoS. Therefore, it is essential to introduce a method capable of handling both common and urgent user requests in a timely manner, which is described in Section 5.

### 4.3. End-to-End Delay

From the IoT users’ end to the destination, each request usually has delay requirements with specific QoS requirements. Considering the end-to-end delay requirements, we divide the delay of incoming requests into the following two parts: urgent-request delay and common-request delay.

**Urgent-Request Delay**: Each request is assumed to disintegrate and can be subdivided into sub-requests, which have to be processed in sequence. For each sub-request, knowledge about available computing resources in the home cloudlet is needed for fair allocation. The decision to allocate resources is based on the availability of computing resources in the related cloudlet; either the available capacity is enough to accommodate the urgent requests, idle instances, or remaining capacity or the request has to be transferred to the adjacent cloudlet if the delay requirements are flexible. However, we prioritize the primary cloudlet. The decision with respect to the allocation of the request at each time slot is denoted by $T_{n}^{u,h}=\left\{1,2,3,\ldots ,T_{n}\right\}$. Therefore, the overall computation delay for users’ urgent requests can be written as follows:

(9) $$d_{n}^{u,h}=\sum_{t\in T_{n}^{u,h}}\;\;\left(d_{qw}^{r^{s}}+d_{f_{l}^{⊕}}^{r^{s}}+d_{exc}^{r^{s}}\right).\sigma t,\;\;\;\;\forall n,t,$$

where $d_{qw}^{r^{s}}$ is the queue waiting delay if $r_{i}^{s}$ is already in processing ($\rho_{i,j}^{t}$), $d_{f_{l}^{⊕}}^{r^{s}}$ is the VNF instance’s activation delay, $d_{exc}^{r^{s}}$ is the request-execution delay, and σt shows the time-slot period.

On the other hand, if the urgent request is assigned to the adjacent cloudlet in time slot *t* due to the unavailability of the required resources and only when the delay requirements are flexible, then the delay includes the transfer delay as well. Therefore, the additional delay for requests waiting in a queue for the adjacent cloudlet can be written as follows:

(10) $$d_{qw^{\prime }}^{r^{s}}=\sum_{t\in T_{n}^{u,ad}}\;\left(d_{tr}^{r^{s}}+d_{exc}^{r^{s}}+d_{f_{l}^{⊕}}^{r^{s}}+d_{\bar{qw}}^{r^{s}}\right),$$

where $d_{tr}^{r^{s}}$ is transmission delay and $d_{\bar{qw}}^{r^{s}}$ is the extra queue waiting delay. Therefore, the overall request delay to the adjacent cloudlet can be denoted as follows:

(11) $$d_{n}^{u,ad}=\sum_{t\in T_{n}^{u,ad}}\sum_{k\in K}b_{k}^{t}.\chi_{n}^{r^{s,t}}\left(\sigma t+\frac{c\left(f_{l}\right).\left(d_{qw^{\prime }}^{r^{s}}+d_{tr}^{r^{s}}+d_{exc}^{r^{s}}\right)}{f_{l}}\right)\;\;\;\;\forall k,n,t,$$

where $\chi_{n}^{r^{s,t}}\in \left\{0,1\right\}$ is a binary decision variable, where $\chi_{n}^{r^{s,t}}=\left\{0\right\}$ when the urgent request is assigned to the home cloudlet and $\chi_{n}^{r^{s,t}}=\left\{1\right\}$ otherwise. $f_{l}$ denotes the available computing resources in the adjacent cloudlet that can be allocated to the transferred request (*n*) if the bandwidth $b_{k}^{t}$ at the *k*th link is enough to transfer the request for processing, and $c\left(f_{l}\right)$ is the computing resource demand of the user at time slot σt.

**Common-Request Delay**: Common requests sent by users are flexible and can be assigned to the home cloudlet or transferred to the adjacent cloudlet. Common requests play an essential role in maintaining a balance of cloudlet resources for allocation. Therefore, the decision with respect to resource allocation at each time slot is depicted by $T_{n}^{c,h}=\left\{1,2,3,\ldots ,T_{n}\right\}$, and the overall computational delay of common requests is illustrated as follows:

(12) $$d_{n}^{c,h}=\sum_{t\in T_{n}^{c,h}}\;1-\chi_{n}^{r^{s,t}}\left(d_{qw}^{r^{s}}+d_{f_{l}^{⊕}}^{r^{s}}+d_{exc}^{r^{s}}\right).\sigma t,\;\;\;\;\forall n,t,$$

where $\chi_{n}^{r^{s,t}}$ is a binary decision variable {0,1}. If $\chi_{n}^{r^{s,t}}=\left\{0\right\}$, the request is processed in the home cloudlet, and $\chi_{n}^{r^{s,t}}=\left\{1\right\}$ otherwise. The terms $d_{qw}^{r^{s}}$, $d_{f_{l}^{⊕}}^{r^{s}}$, and $d_{exc}^{r^{s}}$ represent queue waiting, VNF activation, and request execution delays, respectively. If, due to the limited availability of resources in the home cloudlet or to balance the resource allocation process among cloudlets, a request is transferred to the adjacent cloudlet at time slot *t* [54]; then, such a request can face an overall delay, which includes a processing delay in the home cloudlet, delayed transmission delay to the adjacent cloudlet, and computational delay in the adjacent cloudlet, which can be written as

(13) $$d_{n}^{c,ad}=\sum_{t\in T_{n}^{c,h}}\sum_{k\in K}\varrho_{j,l}^{t}.b_{k}^{t}.\chi_{n}^{r^{s,t}}\left(\sigma t+\frac{c\left(f_{l}\right).\left(d_{qw^{\prime }}^{r^{s}}+d_{tr}^{r^{s}}+d_{exc}^{r^{s}}\right)}{f_{l}}\right),\;\;\;\;\forall j,k,l,n,t,$$

where $\varrho_{j,l}^{t}$ represents the total number of unfinished requests already in processing, which may cause an extra delay and can be released in the next time slot (t+1). $b_{k}^{t}$ denotes the available bandwidth required to transfer the request in the $k^{th}$ link to the adjacent cloudlet. $\chi_{n}^{r^{s,t}}$ is a binary decision variable {0,1}. $p(f_{l})$ shows the computing resource demand of the request at time slot *t*, and $f_{l}$ represents the available computing resources in the cloudlet. $d_{qw}^{r^{s}}$ is the queue waiting delay, $d_{f_{l}^{⊕}}^{r^{s}}$ is the VNF activation delay, and $d_{exc}^{r^{s}}$ is the execution delay on all *k*th links in time slot *t*.

Therefore, the overall delay faced by urgent and common users requests in the home cloudlet ($z_{j}$) or the adjacent cloudlet ($z_{k}$), along with the delay in links, which should not exceed the overall delay requirements ($D_{e}$), can be written as

(14) $$D_{j}^{t}=\sum_{t\in T}^{r_{i}^{s}\in R^{s}}\sum_{k\in K}(d_{n}^{u,h}+d_{n}^{u,ad}+d_{n}^{c,h}+d_{n}^{c,ad})\leq D_{e},\;\;\forall i,j,k,n,t.$$

### 4.4. Energy Consumption Cost and Profit

The network SP accommodates users and charges costs in return by implementing requests on demand. The cost of request implementation is based on the utilization of network resources. We assume that the computing resources are already leased from infrastructure providers and are available to the cloudlets in the form of deactivated VNF instances. To assign a request to a network slice, the VNF instance must be in an activated condition. Activating an instance incurs energy usage, which is converted into the cost of activating and deactivating the VNF instances. This cost is divided into the following two parts: urgent-request cost and common-request cost.

**Urgent-Request Cost**: We consider that some requests sent by IoT users require a rapid response. Let $c(f_{l})$ denote the computing cost of accommodating one unit of traffic ($r_{i}^{s}$) in cloudlet $z_{j}$ during time slot *t*. The cost is determined based on where the request is implemented. In cases where an urgent request ($r_{i}^{s}$) is processed in the home cloudlet ($z_{j}$) and $r_{i}^{s}\leq z_{j}$ with at least one VNF instance ($f_{l}^{⊕}$) activated, the overall cost of energy consumption for urgent requests can be written as

(15) $$c_{n}^{u,h}=\sum_{l=1}^{\left|F\right|}\sum_{t\in T_{n}^{u,h}}\left(c\left(f_{l}\right)+\frac{c\left(f_{l}^{⊕}\right)+c{\left(f_{l}^{⊕}\right)}^{idl}+c{\left(f_{l}^{⊕}\right)}^{pro}+c\left(f_{l}^{⊘}\right)}{f_{l}}\right)\sigma t,$$

where $c(f_{l}^{⊕})$ denotes the cost of activating VNF instances, $c{\left(f_{l}^{⊕}\right)}^{idl}$ is the cost generated by idle VNF instances, $c{\left(f_{l}^{⊕}\right)}^{pro}$ is the processing cost to host requests, $c(f_{l}^{⊘})$ is the cost of deactivating the VNF instance, and σt represents the time period.

When the available resource capacity at $z_{j}$ is not sufficient to fulfill the demand of $r_{i}^{s}$, requests may be transferred to the adjacent cloudlet for processing in the case of flexible requests. Then, the cost ($c(f_{l})$) is charged for the consumption of the bandwidth ($b_{k}^{t}$) at the *k*th link at time slot *t*, and the energy consumption cost to process the user request in the neighboring cloudlets can be denoted as

(16) $$c_{n}^{u,ad}=\sum_{l=1}^{\left|F\right|}\sum_{t\in T_{n}^{u,ad}}\sum_{k\in K}1-\chi_{n}^{r^{s,t}}\left(\sigma t+\frac{c\left(b_{k}^{t}\right)+c\left(f_{l}^{⊕}\right)+c{\left(f_{l}^{⊕}\right)}^{idl}+c{\left(f_{l}^{⊕}\right)}^{pro}}{f_{l}}\right),$$

where $c(b_{k}^{t})$, $c(f_{l}^{⊕})$, $c{\left(f_{l}^{⊕}\right)}^{idl}$, and $c{\left(f_{l}^{⊕}\right)}^{pro}$ represent the bandwidth cost when a request is transferred to the adjacent cloudlet at each time slot (*t*), the cost of activating the VNF instance, the cost of idle VNF instances, and the processing cost, respectively.

**Common-Request Cost**: The cost generated by common requests ($r_{i}^{s}$) allocated to the home cloudlet ($z_{j}$) with acceptable delay requirements can be calculated as follows:

(17) $$c_{n}^{c,h}=\sum_{l=1}^{\left|F\right|}\sum_{t\in T_{n}^{c,h}}\;1-\chi_{n}^{r^{s,t}}\left(\frac{c\left(f_{l}^{⊕}\right)+c{\left(f_{l}^{⊕}\right)}^{idl}+c{\left(f_{l}^{⊕}\right)}^{pro}+c\left(f_{l}^{⊘}\right)}{f_{l}}\right)\sigma t,$$

where $\chi_{n}^{r^{s,t}}$ is the decision variable, $c(f_{l}^{⊕})$ represents the cost of activating VNF instances, $c{\left(f_{l}^{⊕}\right)}^{idl}$ is the cost generated by idle VNF instances, $c{\left(f_{l}^{⊕}\right)}^{pro}$ is the processing cost, $c(f_{l}^{⊘})$ is the cost of deactivating the VNF instance, and σt denotes the time period.

We assume that some common requests arrive without real-time requirements for execution but can also be transferred to the adjacent cloudlet ($z_{j\to k}$) due to their flexible demand, maintaining balance among the cloudlets. Thus, we can express the energy consumption cost of common requests as

(18) $$c_{n}^{c,ad}=\sum_{l=1}^{\left|F\right|}\sum_{t\in T_{n}^{c,ad}}\sum_{k\in K}\chi_{n}^{r^{s,t}}\left(c\left(f_{l}\right)+\frac{c\left(b_{k}^{t}\right)+c\left(f_{l}^{⊕}\right)+c{\left(f_{l}^{⊕}\right)}^{idl}+c{\left(f_{l}^{⊕}\right)}^{pro}+c\left(f_{l}^{⊘}\right)}{f_{l}}\right)\sigma t,$$

where $\chi_{n}^{r^{s,t}}$ is the decision variable to transfer the requests to the adjacent cloudlet at time slot *t*, $c(b_{k}^{t})$ is the bandwidth consumption cost on the links during transfer of requests, $c(f_{l}^{⊕})$ is the VNF activation cost, $c{\left(f_{l}^{⊕}\right)}^{idl}$ is the cost of idle VNF instances, $c{\left(f_{l}^{⊕}\right)}^{pro}$ is the request processing cost, and $c(f_{l}^{⊘})$ is the VNF instance deactivation cost in cases in which it cannot meet the user requirements.

Therefore, the overall cost of energy consumption for the SP to provide the required resources to urgent and common requests in the home cloudlet ($z_{j}$) or an adjacent cloudlet ($z_{k}$) can be written as

(19) $$C_{j}^{t}=\sum_{l=1}^{\left|F\right|}\sum_{t\in T}^{r_{i}^{s}\in R^{s}}\sum_{k\in K}(c_{n}^{u,h}+c_{n}^{u,ad}+c_{n}^{c,h}+c_{n}^{c,ad}).$$

The SP earns profit by charging the cost to provide the required resources for IoT user requests. We assume that $PR^{f_{l}}$ represents the profit obtained by the SP for providing a service instance ($f_{l}$) for a user request at each time slot. The total profit earned by cloudlet $z_{j}$ at each time slot (*t*) is expressed as

(20) $$PR_{j}^{t}=\sum_{r_{i}^{s}\in R^{s}}\tau_{i}.PR_{i}^{f_{l}}.$$

## 5. Optimization Problem for End-to-End Network Slicing

In this section, we illustrate the multi-agent Markov decision process (MAMDP)-based problem formulation and the DRL-based MAgSAC procedure to solve the utility optimization problem in end-to-end network slicing.

### 5.1. MAMDP-Based Problem Formulation

In the proposed multi-agent approach [55], the primary action space is divided into various sub-action spaces, with each agent assigned responsibility for a specific sub-action space. Each agent can observe and analyze the actions of its peer agents. The reward is based on the aggregation of actions from all agents rather than from an individual agent. Therefore, it is crucial for all agents to collaborate to maximize the overall reward, as failure to do so would result in suboptimal outcomes. These actions impact the environment, which subsequently changes.

We present MAMDP as a tuple (N,S,A,U), where N denotes the set of agents, S is the set of possible environment states, A represents the set of actions associated with agents, and U illustrates the utility function. The main objective of the agents is to learn an optimal policy to maximize the accumulated reward at each time slot (*t*). This procedure continues until the agent discovers an optimal policy. A DRL-based approach can quickly map different operating states to actions, in contrast to traditional optimization methods that must solve the problem for every operating state [56]. The end-to-end network slicing problem can be formulated as an MAMDP as follows:

**Agent N:** In the MEC system, each IoT user requests network resources. An agent, represented by N, makes decisions based on available resources. It assigns the required resources to requests in the targeted cloudlet ($z_{j}$) and manages the entire process. These agents ($N_{i}\in N$) are trained to utilize the MAgSAC approach, using their observations of the environment and action space to collectively achieve the optimal policy at each time slot (*t*).

**State Space S:** In each time slot (*t*), the agents observe the status of the MEC environment and aggregate the following information:

- Requests being processed in cloudlet $z_{j}$ at the $t^{th}$ time slot: $\varrho_{j,l}^{t}$, $\forall N_{i}\in N$;
- Available computing capacity of cloudlet $z_{j}$ at the $t^{th}$ time slot: $z_{j}^{cap,t}$, $\forall N_{i}\in N$;
- Idle VNF instances in cloudlet $z_{j}$ at the $t^{th}$ time slot: ${\left(f_{l}^{⊕}\right)}^{idl,t}$, $\forall N_{i}\in N$;
- Active VNF instances in cloudlet $z_{j}$ at the $t^{th}$ time slot: $f_{l}^{⊕,t}$, $\forall N_{i}\in N$;
- Deactivated VNF instances in cloudlet $z_{j}$ at the $t^{th}$ time slot: $f_{l}^{⊘,t}$, $\forall N_{i}\in N$;
- Available bandwidth resources at each edge at the $t^{th}$ time slot: $B_{e,i,j}^{t}$, $\forall N_{i}\in N$.

Therefore, the overall status of the MEC system can be described by state $s_{i}^{t}\in S$, where $s_{i}^{t}=\left(s_{i}^{t},s_{2}^{t},\ldots ,s_{n}^{t}\right)$. This represents the system state of cloudlet *i* at time slot *t*.

(21) $$s_{i}^{t}=\left[r\varrho_{j,l}^{t},\ldots ,z_{j}^{cap,t},\ldots ,{\left(f_{l}^{⊕}\right)}^{idl,t},\ldots ,f_{l}^{⊕,t},\ldots ,f_{l}^{⊘,t},\ldots ,B_{e,i,j}^{t}\right].$$

**Action Space A:** After observing the system state, the agents collaborate to manage the resource allocation process. This includes activating or deactivating necessary VNF instances in the cloudlets to process IoT user requests at each time slot (*t*). The action space (A) is defined as follows: $A=(a_{1},a_{2},\ldots ,a_{n})$, which encompasses the following actions in the observed environment:

- Amount of computing resources in cloudlet $z_{j}$ that can be assigned to a VNF instance at the $t^{th}$ time slot: $f_{l}^{t}$, $\forall N_{i}\in N$;
- Activation of a VNF instance in cloudlet $z_{j}$ to fulfill user requirements at the $t^{th}$ time slot: $f_{l}^{⊕,t}$, $\forall N_{i}\in N$;
- Amount of remaining computing resources in cloudlet $z_{j}$ that can still be assigned at the $t^{th}$ time slot: $z_{j}^{*cap,t}$, $\forall N_{i}\in N$;
- Deactivation of a VNF instance in cloudlet $z_{j}$ at the $t^{th}$ time slot: $f_{l}^{⊘,t}$, $\forall N_{i}\in N$;
- Transfer of the request to the adjacent cloudlet ($z_{i}$) at the $t^{th}$ time slot: $z_{i\to j}^{t}$, $\forall N_{i}\in N$;

Therefore, the action space ($a_{i}^{t}\in A$) of the agents in the $i^{th}$ cloudlet at time slot *t* can be written as

(22) $$a_{i}^{t}=\left[a_{f_{l}^{t}},\ldots ,a_{f_{l}^{⊕,t}},\ldots ,a_{z_{j}^{*cap,t}},\ldots ,a_{f_{l}^{⊘,t}},\ldots ,a_{z_{i\to j}^{t}}\right]$$

While taking actions to satisfy user requirements, the following constraints must also be considered:

- One VNF instance must be activated in the cloudlet to assign the user request;
- If an idle instance is available, it must meet the needs of the user request to promote reuse;
- The remaining capacity in the cloudlet for the instance should be greater than the incoming request;
- To transfer the request to an adjacent cloudlet, the bandwidth available between the two cloudlet edges ($e_{i}\leftarrow e_{j}$) should be sufficient for this transfer.

When deciding whether to activate or deactivate the VNF instance for assignment, it is necessary to keep track of unfinished requests. Thus, the remaining capacity in cloudlet $z_{j}$ that can still be allocated at time slot *t* is expressed as $z_{j}^{*cap,t}=z_{j}^{cap}-\sum_{l=1}^{\left|F\right|}\varrho_{j,l}^{t}\cdot p\left(f_{l}\right)$. Then, the decision with respect to the assignment of resources for action $a_{i}^{t}$ is further specified by $\phi_{j,l}^{t}=\left\lfloor \frac{z_{j}^{*cap,t}\cdot {\overset{‘}{a}}_{j}}{p(f_{l})}\right\rfloor$.

**Utility Function U:**

With 5G network slicing, the resource demands of IoT users can vary greatly. Because different services, from security cameras to online gaming, have distinct utility functions, they prioritize different network aspects. For example, a weather sensor might prioritize low latency (fast response) for timely data transmission, while a video call might prioritize high bandwidth for smooth video streaming. Well-defined utility functions enable agents to make well-informed judgments on the most effective way to utilize resources, resulting in the optimal distribution of vital resources. In utility optimization functions, our goal is to optimize cloudlet resources intelligently while allocating them to IoT user requests with minimum latency. Intelligent allocation involves effectively optimizing overall utility by intelligently activating and deactivating VNF instances, reutilizing idle instances and remaining capacity, efficiently utilizing bandwidth during request transfer, and maintaining QoS according to user demand and MEC network states.

For each IoT user request ($r_{i}^{s}$), the agent ($N_{i}$) takes an action ($a_{i}^{t}$) based on the given environmental state ($s_{i}^{t}$), and the environment responds with the immediate utility. The utility of the MEC network is based on revenue and cost. Revenue comprises efficient utilization of MEC resources, considering QoS, while the cost includes the energy consumption required to accommodate IoT users. According to Equations (19) and (20), after excluding the cost from the revenue ($PR_{j}^{t}-C_{j}^{t}$), the immediate utility function of cloudlet $z_{j}$ of the $N_{i}$ at each time (*t*) can be presented as

(23) $$U_{i}^{t}=\sum_{t=1}^{T}\sum_{j=1}^{\left|Z\right|}\left(PR_{j}^{t}-C_{j}^{t}\right).$$

Based on the utility model ($u_{i}^{t}\in U_{i}^{t}$) defined above, we can express the optimization problem in the following way:

(24) $$\underset{{\left(f_{l},f_{l}^{⊕},z_{j}^{*cap},f_{l}^{⊘},z_{i\to j}\right)}^{t}}{\mathrm{Maximize}}\lim\underset{T\to \infty }{\sup}E\left[\sum_{t=0}^{T-1}U_{i}^{t},\right]$$

subject to

(24a) $$\sum_{l=1}^{\left|F\right|}f_{l}^{t}\;.\;p\left(f_{l}\right)\leq z_{j}^{cap},\;\;\forall j,l,t$$

(24b) $$\sum_{l=1}^{\left|F\right|}f_{l}^{t}\;.\;p\left(f_{l}\right)\leq z_{j}^{*cap},\;\;\forall j,l,t$$

(24c) $$\sum_{l=1}^{\left|F\right|}f_{l}^{t}\;.\;p\left(f_{l}\right)\;+\sum_{r_{i}^{s}\in R^{s,t}}f_{l}^{⊕}\;.\;p\left(f_{l}\right)\leq z_{j}^{cap},\;\;\forall i,j,l,t$$

(24d) $$\sum_{f_{l}\in F}{\left(f_{l}^{⊕}\right)}^{idl}\leq f_{l}^{t}-\varrho_{j,l}^{t},\;\;\forall j,l,t$$

(24e) $$\varrho_{j,l}^{t}\leq f_{l}^{t},\;\;\forall j,l,t$$

(24f) $$\sum_{t=1}^{T}\sum_{j=1}^{\left|Z\right|}\chi_{n}^{r^{s,t}},\;\;\forall j,n,t$$

(24g) $$f_{l}^{t}\geq 0,\;\;\forall l,t$$

(24h) $$\varrho_{j,l}^{t}\in Z,\;\;\forall j,l,t$$

(24i) $$\chi_{n}^{r^{s,t}}\in \left\{0,1\right\}\;\;\forall n,t$$

(24j) $$\chi_{n}^{r^{s,t}}{\left(f_{l}^{⊕}\right)}^{idl}\in \left\{0,1\right\}\;\;\forall l,n,t$$

(24k) $$\chi_{n}^{r^{s,t}}\left(f_{l}^{⊕}\right)\in \left\{0,1\right\}\;\;\forall l,n,t$$

(24l) $$\chi_{n}^{r^{s,t}}\left(f_{l)}^{⊘}\right)\in \left\{0,1\right\}\;\;\forall l,n,t$$

where E[.] represents an estimate of the agent’s long-term utility. Constraint (24a) ensures that the computing demand of the user for resources at each time slot does not exceed the total amount of available capacity in the related cloudlet. Constraint (24b) indicates that the computing demand of the user does not exceed the remaining available capacity in the cloudlet. Constraint (24c) serves as a preventive measure to ensure that the closest cloudlets do not exceed their computational capabilities by enabling an excessive number of VNF instances. The initial part of constraint (24d) denotes the total number of requests that demand a VNF instance ($f_{l}$) and that are assigned to an idle instance in cloudlet $z_{j}$. This total must not exceed the threshold stated in the second part of the constraint. The second portion of the constraint refers to the number of idle VNF instances that are currently available. Constraint (24e) denotes the number of VNF instances, which is equal to or greater than the number of instances being processed. This is essential for efficient resource allocation, since the scaling of idle instances should be minimized. Constraint (24f) guarantees that each user request is solely assigned to a single cloudlet—either the home cloudlet or an adjacent cloudlet.

### 5.2. Multi-Agent Soft Actor–Critic-Based Learning

**Figure 2:**
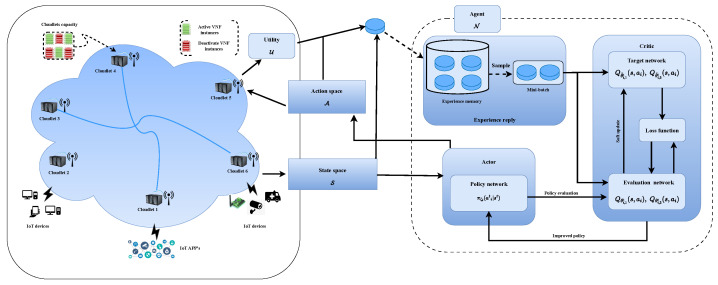
DRL-based multi-agent SAC System.

We adopt a model-free, SAC-based multi-agent DRL strategy to execute IoT requests within the MEC network by modifying the standard reinforcement learning (RL) approach. Figure 2 illustrates the multi-agent SAC-based architecture. The multi-agent SAC framework comprises three primary components aimed at enhancing the performance of the approach [57].

1. **Actor–Critic Structure:** SAC operates according to the actor–critic structure, which includes an actor part and a critic part. The actor contributes to determination of the optimal strategy that maximizes expected utility, whereas the critic provides an estimation of the state and state–action value over that period. By leveraging the actor–critic structure, SAC effectively combines policy-based and value-based RL, which is a positive aspect of this approach.
2. **Entropy Maximization:** By incorporating entropy assessments of policies into the utility function, the stochasticity of SAC’s policy substantially improves, thereby enabling the exploration of a wider range of potentially optimal decisions. Compared to previous policy-based DRL methods, the SAC approach demonstrates greater adaptability and scalability, allowing it to adapt effectively in stochastic environments. In short, maximizing entropy in the SAC algorithm promotes exploration and enhances the ability of the policy to adapt to complex and extremely large environments.
3. **Off-Policy Learning:** To train network parameters based on the experience replay strategy, SAC utilizes an off-policy formulation. This approach enables the efficient utilization of sampled experiences to achieve smooth convergence. SAC leverages the following three key features: off-policy learning, the actor–critic framework, and entropy maximization. These features collectively contribute to SAC’s effectiveness in continuous control actions.

Following this, we offer a detailed description of how the SAC strategy is applied during the learning process. Initially, we delve into the concept of soft value functions within the SAC framework. Then, we explain the specific learning processes of both the critic (policy assessment) and the actor (policy improvement).

#### 5.2.1. Soft Value Function

Our approach aims to find a policy ($\pi_{i}\left(a_{i}|s\right)$) that maximizes the expected utility in the long run. In the SAC-based DRL technique [58], an entropy term ($H\left(\pi_{i}\left(a_{i}|s\right)\right)=-\log\left(\pi_{i}\left(a_{i}|s\right)\right)$) is included in the utility function to ensure continuous exploration of the environment. The objective related to maximizing entropy is known as the entropy objective function and can be defined as follows:

(25) $$\pi_{i}^{*}=\arg\max_{\pi_{i}}\underset{s^{t},a_{i}^{t}∼\pi_{i}}{E}\left[\sum_{t=0}^{\infty }\gamma^{t}\left(u_{i}^{t}+\alpha_{i}H\left(\pi_{i}\left(\cdot |s^{t}\right)\right)\right)\right],$$

where the function H(·) is used to compute the entropy of policy $\pi_{i}$, represented as $H\left(\pi_{i}\left(\cdot |s\right)\right)=E_{a_{i}∼\pi_{i}}\left[-\log(\pi_{i}\left(a_{i}|s\right))\right]$. The symbol γ denotes the discount factor used in long-term utility calculation. Additionally, $\alpha_{i}$ functions as the temperature parameter that controls the degree of entropy regularization. It serves as a learning parameter crucial for achieving an optimal balance between exploration and exploitation actions.

The soft state-value function enables the determination of how profitable state *s* is, considering the expected return. The soft Q-value function describes the long-term benefit of action $a_{i}^{t}$ in state *s*, facilitating the analysis and improvement of policy $\pi_{i}$ during training. The soft state-value function integrates the expected return augmented by entropy, formulated as follows:

(26) $$V_{i}^{\pi }\left(s^{t}\right)=\underset{s^{t},a_{i}^{t}∼\pi_{i}}{E}\left[\sum_{t=0}^{\infty }\gamma^{t}\left(u_{i}^{t}+\alpha_{i}H\left(\pi_{i}\left(\cdot |s^{t}\right)\right)\right)|s_{0}=s^{t}\right].$$

Therefore, the soft Q function can be expressed similarly as

(27) $$\begin{array}{c} Q_{i}^{\pi }\left(s^{t},a_{i}^{t}\right)=\underset{s^{t},a_{i}^{t}∼\pi_{i}}{E}\left[\sum_{t=0}^{\infty }\gamma^{t}u_{i}^{t}+\alpha_{i}\sum_{t=1}^{\infty }\gamma^{t}H\left(\pi_{i}\left(\cdot |s^{t}\right)\right)|s_{0}=s^{t},a_{i,0}^{t}=a_{i}^{t}\right]. \end{array}$$

SAC utilizes the provided policy ($\pi_{i}$) to iteratively compute the soft value functions. The relationship between the soft state-value function ($V_{i}^{\pi }\left(s^{t}\right)$) and the soft Q function ($Q_{i}^{\pi }\left(s^{t},a_{i}^{t}\right)$) is presented in accordance with the Bellman equation as follows:

(28) $$\begin{array}{cc} Q_{i}^{\pi }\left(s^{t},a_{i}^{t}\right) & =u_{i}^{t}+\underset{s^{t+1}∼\pi_{i}}{E}\left[V_{i}^{\pi }\left(s^{t+1}\right)\right], \end{array}$$

(29) $$\begin{array}{cc} V_{i}^{\pi }\left(s^{t}\right) & =\underset{s^{t},a_{i}^{t}∼\pi_{i}}{E}\left[Q_{i}^{\pi }\left(s^{t},a_{i}^{t}\right)+\alpha H\left(\pi_{i}\left(\cdot |s^{t}\right)\right)\right]. \end{array}$$

Employing DNNs in resource optimization challenges allows the algorithm to precisely represent the soft action-value function, considering the complex state and action space. This capability is effective in handling a large part of the problem space and improving decision making. Such a feature enhances the algorithm’s ability to navigate the complexities of resource allocation, ultimately optimizing resource consumption and QoS efficiency [59].

Afterwords, fully a connected DNN is employed with the parameters of $\theta_{i}$ and $\zeta_{i}$, the soft action-value function of $Q_{i}^{\pi }\left(s^{t},a_{i}^{t}\right)$, and the policy of $\pi_{i}\left(a_{i}^{t}|s^{t}\right)$, which can also be parameterized as $Q_{\theta_{i}}\left(s^{t},a_{i}^{t}\right)$ and $\pi_{\zeta_{i}}\left(a_{i}^{t}|s^{t}\right)$, respectively.

#### 5.2.2. Policy Evaluation

During training, the experience replay strategy is used to disrupt the temporal correlations of samples. The performed actions, the transition of network environment states in each time slot (*t*), and the achieved immediate utility can be represented by a tuple $\left\{s^{t},a_{i}^{t},u_{i}^{t},s^{t+1}\right\}$. These tuples are stored in a fixed-size experience replay buffer ($B_{r}$). Subsequently, the actor and critic are updated by randomly sampling a mini batch ($B_{\varpi }$) of tuples (${\left\{s^{t,b},a_{i}^{t,b},u_{i}^{t,b},s^{t+1,b}\right\}}_{N_{i}\in N}$) from this buffer.

To mitigate the overestimation of state values, two separate critic neural networks with parameters of $\theta_{i}^{1}$ and $\theta_{i}^{2}$ are employed. The smaller value between $Q_{\theta_{i}^{1}}$ and $Q_{\theta_{i}^{2}}$ is selected as the actual Q value. Each evaluation critic network parameter ($\theta_{i}^{\iota },\forall \iota \in \left\{1,2\right\}$) is updated individually by minimizing the loss function ($L(\theta_{i}^{\iota })$). To reduce positive bias in policy improvement, a target soft action-value function with parameters pf ${\tilde{\theta }}_{i}^{\iota }$ is introduced. The parameters of the target networks, denoted as ${\tilde{\theta }}_{i}^{\iota }$, are regularly updated by blending the current parameters with a fraction of the previous target parameters. This approach enhances training stability by providing more consistent target values to the critic networks. The target soft action-value function, represented by ${\tilde{\theta }}_{i}^{\iota }$, is then expressed as follows:

(30) $$\begin{array}{cc} \ell_{i}^{t,b} & =u_{i}^{t,b}+\gamma (\min_{\iota =1,2}Q_{{\tilde{\theta^{\iota }}}_{i}}\left(s^{t+1,b},a_{i}^{t+1,b}\right)\\ & -\alpha_{i}\log\left(\pi_{\zeta_{i}}\left(a_{i}^{t+1,b}|s^{t+1,b}\right)\right)). \end{array}$$

After that, the critic loss function can be stated as

(31) $$£\left(\theta_{i}^{\iota }\right)=\frac{1}{2B_{\varpi }}\sum_{b=1}^{B_{\varpi }}{\left(\min_{\iota =1,2}Q_{\theta_{i}^{\iota }}\left(s^{t,b},a_{i}^{t,b}\right)-\ell_{i}^{t,b}\right)}^{2}.$$

Therefore, after updating the critic networks, we can find the stochastic gradient as follows:

(32) $$\begin{array}{cc} \; & \nabla_{\theta_{i}^{\iota }}£\left(\theta_{i}^{\iota }\right)=\frac{1}{B_{\varpi }}\sum_{\iota =1}^{B_{\varpi }}\nabla_{\theta_{i}^{\iota }}Q_{\theta_{i}^{\iota }}\left(s^{t,b},a_{i}^{t,b}\right)\\ & \;\cdot (Q_{\theta_{i}^{\iota }}\left(s^{t,b},a_{i}^{t,b}\right)-\left(u_{i}^{t,b}+\gamma (Q_{{\tilde{\theta }}_{i}^{\iota }}(s^{t+1,b},a_{i}^{t+1,b}\right)\\ & \;-\alpha_{i}\log\left(\pi_{\zeta_{i}}\left(a_{i}^{t+1,b}|s^{t+1,b}\right)\right)))). \end{array}$$

Additionally, to ensure the stability of the learning procedure, the parameters of the target critic network (${\tilde{\theta }}_{i}^{\iota }$) are updated based on the parameters of the evaluation critic network ($\theta_{i}^{\iota }$) using a soft-updating approach, which is expressed as follows:

(33) $$\tilde{\theta_{i}^{\iota }}=\vartheta \theta_{i}^{\iota }+\left(1-\vartheta \right)\tilde{\theta_{i}^{\iota }},\forall \iota =1,2,$$

where ϑ∈(0,1) indicates the update factor.

#### 5.2.3. Policy Improvement

The goal is to operate the model at a lower cost while maximizing total utility within a given time frame through policy optimization. Conversely, an inferior current policy may lead to lower training accuracy or generate significant costs, ultimately reducing utility. Therefore, enhancing the policy is crucial. Iteratively refining the policy parameters ensures continual progress towards improved effectiveness and efficiency. Ongoing policy improvement also enhances the model’s adaptability to evolving environments and data variations, facilitating effective decision making and ensuring sustained long-term improvements. The policy parameters are refined by minimizing the expected Kullback–Leibler (KL) divergence in the algorithm as follows:

(34) $$J\left(\zeta_{i}\right)=\underset{s^{t}∼B_{\varpi }}{E}\left[D_{\mathrm{KL}}\left(\pi_{\zeta_{i}}\left(\cdot |s^{t}\right)||\frac{\exp\left(\min_{\iota =1,2}Q_{\theta_{i}^{\iota }}\left(s^{t},\cdot \right)\right)}{\Upsilon_{\theta_{i}^{\iota }}\left(s^{t}\right)}\right)\right],$$

where the difference between the distributions ($\pi_{\zeta_{i}}\left(\cdot |s^{t}\right)$ and $\frac{\exp\left(\min_{\iota =1,2}Q_{\theta_{i}^{\iota }}\left(s^{t},\cdot \right)\right)}{\Upsilon_{\theta_{i}^{\iota }}\left(s^{t}\right)}$) is measured by the KL divergence. $\Upsilon_{\theta_{i}^{\iota }}\left(s^{t}\right)$ is a constant partition function that does not affect the updated policy gradient. Therefore, by scaling with the temperature parameter ($\alpha_{i}$) and removing the term $E_{a^{t}∼\pi_{\zeta }}\left[\alpha_{i}\log\Upsilon_{\theta_{i}^{\iota }}\left(s^{t}\right)\right]$, the KL divergence can be reformulated as follows:

$$J\left(\zeta_{i}\right)=-\underset{s^{t+1}∼B_{\varpi }}{E}[\underset{a^{t+1}∼\pi_{\zeta_{i}}}{E}\left[\min_{\iota =1,2}Q_{\tilde{\theta_{i}^{\iota }}}\left(s^{t+1,b},a_{i}^{t+1,b}\right)\right.$$

(35) $$-\alpha_{i}\log(\pi_{\zeta_{i}}(a_{i}^{t+1,b}|s^{t+1,b}))]].$$

In this context, $\delta_{\zeta_{i}}\left(\varepsilon^{t};s^{t}\right)$ represents the neural network transformation used to reparameterize the policy, where $\varepsilon^{t}$ denotes an input noise sample from a Gaussian distribution. Thus, we can rewrite Equation (30) as

$$J\left(\zeta_{i}\right)=-\underset{s^{t+1}∼B_{\varpi },\varepsilon^{t}∼\pi_{\zeta_{i}}}{E}[\min_{\iota =1,2}Q_{{\tilde{\theta }}_{i}^{\iota }}\left(s^{t+1,b},\delta_{\zeta_{i}}\left(\varepsilon^{t};s^{t,b}\right)\right)$$

(36) $$-\alpha_{i}\log(\pi_{\zeta_{i}}(\delta_{\zeta_{i}}(\varepsilon^{t};s^{t,b})|s^{t+1,b}))].$$

Therefore, we can obtain an approximate representation of the policy gradient as follows:

(37) $$\begin{array}{cc} \nabla_{\zeta_{i}}J\left(\zeta_{i}\right)= & \nabla_{\zeta_{i}}\alpha_{i}\log\left(\pi_{\zeta_{i}}\left(a_{i}^{t,b}|s^{t,b}\right)\right)\\ & +\left(\nabla_{\zeta_{i}}\alpha_{i}\log\left(\pi_{\zeta_{i}}\left(a_{i}^{t,b}|s^{t,b}\right)\right)\right.\\ & -\left.\nabla_{\zeta_{i}}Q\left(s^{t,b},a_{i}^{t,b}\right)\right)\nabla_{\zeta_{i}}\delta_{\zeta_{i}}\left(\varepsilon^{t};s^{t,b}\right). \end{array}$$

The learning process alternates between soft policy evaluation and soft policy improvement until convergence towards the optimal policy with maximum entropy is achieved. This iterative cycle maintains policy flexibility and exploratory capability, enabling adaptation to various scenarios and the unpredictability of the environment. By increasing entropy, the policy strikes a balance between exploration and exploitation, facilitating the exploration of the state–action space while avoiding premature convergence to sub-optimal solutions.

After minimizing the loss function, according to Equations (31) and (36), the gradient can be calculated to update the temperature parameters automatically as follows:

(38) $$J\left(\alpha_{i}\right)=\underset{a^{t,b}∼\pi_{\theta_{i}}}{E}\left[-\alpha_{i}\log\left(\pi_{\theta_{i}}\left(a^{t,b}|s^{t,b}\right)\right)-\alpha_{i}\bar{H}\right],$$

where $a^{t,b}=\left\{a_{i}^{t,b}\right\}$ describes the set of actions and $\bar{H}$ denotes the target entropy value.

### 5.3. Detailed Examination of Algorithms

The primary goal of Algorithm 1 is to identify resources in the cloudlet where IoT user requests arrive, assess the nature of each request, and allocate resources accordingly. Upon the arrival of a request in the cloudlet during each time slot, it searches within the set of cloudlets in the MEC for activated VNF instances that closely match the request requirements, including latency constraints. If the request is not common and requires urgent execution, it locates an activated VNF instance in the cloudlet with sufficient capacity to meet the request’s requirements. Alternatively, it searches for an idle VNF instance in the home cloudlet that has completed its processing and is ready for deactivation or has remaining capacity. If the latency requirements are flexible, it explores activated instances in adjacent cloudlets. Otherwise, it activates a new VNF instance that precisely matches the requirements to minimize resource waste. Urgent requests are prioritized to the primary cloudlets, and to balance resource allocation among cloudlets, common requests may be transferred to other cloudlets.
**Algorithm 1** Request Assignment**Input:** A set of cloudlets Z at the MEC network G=(Z∪V,E) and at each time slot *t* a set of IoT users requests $R^{s}$.**Output:** Find the related cloudlet $z_{j}$ to assign the request $r_{i}^{s}$ at each time slot *t*.
1:**for** $i=1,2,\cdots ,|R^{s,t}|$ **do**2:   **for** j=1,2,⋯,|Z| **do**3:     Find the set of cloudlets $z_{j}$ which have activated VNF instances $f_{l}^{⊕}$ and that instance capacity matches the requirements of $r_{i}^{s}$ along with delay $D_{e}$ requirements.4:     **if** $r_{urg}^{s}>1$ **then**5:        if $f_{l}^{⊘}=1$, find the $f_{l}^{⊕}$ in $z_{j}$ with $z_{j}^{cap}-\sum_{l=1}^{\left|F\right|}\varrho_{j,l}^{t}.p\left(f_{l}\right)$ still meet the demand of the $r_{urg}^{s}$, or any idle instance ${\left(f_{l}\right)}^{idl}$ already finished computing reutilize it, and if delay requirements assign to $z_{i\to j}$, otherwise initialize a new $f_{l}^{⊕}$ with exact requirement at $z_{j}\in Z$ and assign $r_{urg}^{s}$ to it.6:        **if** $r_{urg}^{s}\leq 1$ **then**7:          At any $z_{j}\in Z$ find a $f_{l}^{⊕}$ with residual capacity or ${\left(f_{l}^{⊕}\right)}^{idl}$.8:        **else**9:          Transfer $r_{i}^{s}$ to the $z_{i\to j}$.10:return The decision of requests $r_{i}^{s}$ assignment at cloudlet $z_{j}$.


Algorithm 2 presents an online soft actor–critic (SAC) mechanism for resource allocation to requests in cloudlets. Each agent in this approach takes system states as inputs and employs an actor network to generate policies for decision making. A critic network assesses the quality of these actions. The parameters of the actor network are updated using policy gradients. This process facilitates decisions on resource assignments based on the available capacity of VNF instances in the cloudlets, ensuring efficient resource allocation.
**Algorithm 2** Online Soft Actor–Critic-based Process to Assign Resources**Input:** The system states $s_{i}^{t}=\left(r\varrho_{j,l}^{t},z_{j}^{cap,t},{\left(f_{l}^{⊕}\right)}^{idl,t},f_{l}^{⊕,t},f_{l}^{⊘,t},B_{e,i,j}^{t}\right)$, the actor network with the parameter $\theta_{i}$ and the critic network with the parameter $\zeta_{i}$ for each agent $N_{i}$.**Output:** The decision of resource assignment at the cloudlet $z_{j}$.
1:The given system states $s_{i}^{t}$ as input, the output of the actor network is $\pi_{i}\left(a_{i}^{t}|s^{t};\theta_{i}\right)$ for each agent $N_{i}$;2:Select sample action $s_{i}^{t}$ based on the distribution $\pi_{i}\left(a_{i}^{t}|s^{t};\theta_{i}\right)$ for each agent $N_{i}$;3:By using the critic network evaluate the action-value function $Q_{i}\left(s^{t},a_{i}^{t};\zeta_{i}\right)$ for each agent $N_{i}$;4:Update the actor network parameters $\theta_{i}$ by calculating the policy gradient for each agent $N_{i}$;5:$\phi_{j,l}^{t}=\left\lfloor \frac{z_{j}^{*cap,t}.{\overset{‘}{a}}_{j}}{p(f_{l})}\right\rfloor$, ∀l∈{1,2,3,…,|F|};6:return The decision $\phi_{j,l}^{t}$ of resource assignment to $R^{s}$ at cloudlet $z_{j}$.


The main steps of our proposed DRL-based MAgSAC algorithm are illustrated here. In the first stage, the parameters of the soft Q-value functions ($Q_{\theta_{i}}^{\iota }\left(s^{t},a_{i}^{t}\right),\forall \iota \in \left\{1,2\right\}$) are initialized with weights of $\theta_{i}^{\iota }$. Subsequently, the target parameters (${\tilde{\theta }}_{i}^{\iota }$) of the soft Q-value functions are initialized with the same weights ($\theta_{i}^{\iota }$). Following this, the policy parameters ($\pi_{\zeta_{i}}\left(a_{i}|s\right)$) are initialized with random weights, and experiences are sampled from the experience replay memory ($B_{r}$).

In the second stage, within each cloudlet and episode, agents observe the initial state ($s^{0}$) of the mobile edge computing environment. In the third stage, at each time slot, each agent observes the network state( $s_{i}^{t}$); then, the actor takes action $a_{i}^{t}$ based on the observed state ($s_{i}^{t}$) and the defined policy ($\pi_{\zeta_{i}}\left(a_{i}^{t}|s^{t}\right)$). The decision to allocate resources according to the requirements is made by invoking Algorithm 2, while Algorithm 1 predicts the nature of the requests during the assignment stage. After taking actions in the environment, each agent receives feedback in the form of utility $u_{i}^{t}$, and the network environment transitions from $s^{t}$ to $s^{t+1}$.

In the fourth stage, the experiences of agent actions in the environment are stored in the experience replay memory ($B_{r}$) as tuples $\left\{s^{t},a_{i}^{t},u_{i}^{t},s^{t+1}\right\}$. In the fifth stage, a mini batch ($B_{\varpi }$) of experiences is randomly selected from the experience replay memory ($B_{r}$), and the soft Q-value parameters ($\theta_{i}^{\iota }$) and policy parameters ($\zeta_{i}$) are updated by minimizing loss functions $£\left(\theta_{i}^{\iota }\right)$ and $J(\zeta_{i})$ according to Equations (31) and (36), respectively. The gradients can be calculated automatically using Equation (38); then, the temperature parameters are updated.

In the last stage, the parameters of the target critic network (${\tilde{\theta }}_{i}^{\iota }$) are updated based on the assessment of the critic network parameters ($\theta_{i}^{\iota }$) according to Equation (33).

The algorithm, referenced as Algorithm 3, employs an online SAC approach to assign requests to suitable cloudlets and allocate resources accordingly. Actions related to request assignment and resource allocation in the cloudlets are facilitated by invoking Algorithms 1–4.
**Algorithm 3** Online Soft Actor–Critic-Based Algorithm to Assign Requests and Resource Allocation**Input:** A set of cloudlets Z with VNF instances F at the MEC network G=(Z∪V,E) and at each time slot *t* a set of IoT users requests $R^{s}$.**Output:** The decision of request $r_{i}^{s}$ assignment and resource allocation at each time slot *t*.
1:Train the actor and critic networks by apply MAgSAC Algorithm 4;2:**for** t=1,2,…,T **do**3:   **for** i=1,2,…,Z **do**4:     Invoke the Algorithm 2 to allocate the resources by deciding $\phi_{j,k}^{t}$ to accommodate the $r_{i}^{s}$ the VNF instances $f_{l}\in F$ will be activate $f_{l}^{⊕}$ or deactivate $f_{l}^{⊘}$ at the cloudlet $z_{j}$;5:   To assign the requests $r_{i}^{s}$ and $r_{urg}^{s}$ invoke the Algorithm 1 and calculate the $f_{l}^{⊕}$, ${\left(f_{l}^{⊕}\right)}^{idl}$ at each time slot *t* and update the system according;6:returrn The decision of the request assignment and resource allocation after action $a_{i}^{t}$ with $z_{j}^{*cap},{\left(f_{l}^{⊕}\right)}^{idl},f_{l}^{⊕}.$


**Algorithm 4** Training of MAgSAC
**Input:** A set of cloudlets Z with VNF instances F at the MEC network G=(Z∪V,E) and at each time slot *t* a set of IoT users requests $R^{s}$.**Output:** The trained networks of updated policy $a=a_{1},a_{2},\ldots a_{n}$
1:Initialize the critic network with parameters $\theta_{i}^{\iota }$(ι=1,2), initialize the target critic network with parameters ${\tilde{\theta }}_{i}^{\iota }$ = $\theta_{i}^{\iota }$, Initialize the actor network with parameters $\zeta_{i}$, Initialize the temperature parameter $\alpha_{i}$, and experience reply memory $B_{r}$ for each agent $N_{i}$.2:**for** i=1,2,…,|Z| **do**3:   **for** each episode = 1 **do**4:     Initialize the state $s^{0}=\left\{s_{i}^{0}\right\}$ for all agents N;5:     **for** t=1,2,…,T **do**6:        **for** each agent N **do**7:          Observer the system states $s_{i}^{t}=\left(r\varrho_{j,l}^{t},z_{j}^{cap,t},{\left(f_{l}^{⊕}\right)}^{idl,t},f_{l}^{⊕,t},f_{l}^{⊘,t},B_{e,i,j}^{t}\right)$;8:          Take the decision with action $a_{i}^{t}=\left(a_{f_{l}^{t}},a_{f_{l}^{⊕,t}},a_{z_{j}^{*cap,t}},a_{f_{l}^{⊘,t}},a_{z_{i\to j}^{t}}\right)$ for all N by following the policy $\pi_{\zeta_{i}}\left(a_{i}^{t}|s^{t}\right)$;9:          By invoking the Algorithm 2 find the $\phi_{j,l}^{t}=\left\lfloor \frac{z_{j}^{*cap,t}.{\overset{‘}{a}}_{j}}{p(f_{l})}\right\rfloor$, ∀k∈{1,2,3,…,|F|};10:         At the request assignment stage call the Algorithm 1 for common $r^{s}$ and urgent requests $r_{urg}^{s}$;11:         Aggregate the positive reward of the $z_{j}$ at each time slot *t* by following the Equation (20);12:         Observe the utility $u_{i}^{t}$ and get the next state $s^{t+1}$;13:         Store these tuples $\left\{s^{t},a_{i}^{t},u_{i}^{t},s^{t+1}\right\}$ in $B_{r}$ replace the oldest experience;14:         A mini-batch of $B_{\varpi }$ experience $\left\{s^{t},a_{i}^{t},u_{i}^{t},s^{t+1}\right\}$ randomly selected from $B_{r}$;15:         Calculate the target Q-value $\ell_{i}^{t,b}$ from Equation (30);16:         Minimize the loss function from Equation (31) by updating the parameters $\theta_{i}^{\iota }$ of soft Q-value function;17:         Update the policy parameters $\zeta_{i}$ from the gradient in Equation (37);18:         Temperature parameter $\alpha_{i}$ update by computing the gradient from Equation (38);19:         Update the target networks $\theta_{i}^{\iota }$ with Equation (33);



## 6. Complexity Analysis

In the initialization phase (Line 1), the critic and actor networks, temperature parameter, and experience replay memory are initialized for each agent, which runs in constant time O(1). In the main loop (Lines 2–6), there are three nested loops; the first loop iterates over the number of cloudlets (T(Z)), the second and fourth loops iterate over the number of agents (T(n)), and the third loop iterates over the number of time slots (T(t)). This gives a combined complexity of $O(T\left(Z\right)\cdot T{\left(n\right)}^{2}\cdot T\left(t\right))$. The subsequent steps (Lines 7–19) are constant time operations (O(1)), as they involve updating parameters using mini-batch experience, which is performed in constant time. Therefore, the overall time complexity of the algorithm is $O\left(1\right)+O\left(T\left(Z\right)\cdot T{\left(n\right)}^{2}\cdot T\left(t\right)\right)+O\left(1\right)=O\left(T\left(Z\right)\cdot T{\left(n\right)}^{2}\cdot T\left(t\right)\right)$.

## 7. Results and Discussion

In this section, we conduct comprehensive simulations to assess the efficiency of our proposed approach in mobile edge computing scenarios. Additionally, we evaluate the influence of significant parameters on the effectiveness of the proposed technique.

### 7.1. Parameter Setup

The simulations are performed on a Dell Core i7-9850H CPU @ 3.00 GHz (12 CPUs) with an Intel UHD 630 graphics card, NVIDIA Quadro T2000, and 64GB RAM running on a Windows 11 Pro 64-bit operating system. The experimental parameters are set in accordance with the research work reported in [60,61,62]. In an MEC network, we consider a network size ranging from 10 to 200 nodes; each topology is generated using GT-ITM [63], and the number of cloudlets is set to 20% of the network size. The computing capacity of each cloudlet ranges from 40,000 to 120,000 MHs [64], and the bandwidth of each link varies from 30 Mbps to 100 Mbps [65]. The computing resource requirements for five types of network functions—firewall, NAT, collision detector, and IDS—are adopted from [66,67]. Each network slicing request is randomly generated from 10 to 100 Megabytes, and the delay requirements for common and urgent requests range from 1 to 20 ms [68]. We utilize randomly generated networks ranging from 10 to 200 nodes to train our model. Subsequently, we employ the trained instances for examination on networks with varied sizes and parameter variations to demonstrate the model’s adaptability. The resulting values of the proposed algorithm in each figure represent the mean values, and the algorithm’s running time depends on the machine. These parameter settings are adopted in our experimental analysis unless otherwise specified.

We present a comparative analysis of the performance of our proposed MAgSAC algorithm with that of the following six benchmark approaches:

- *MAA3C-based approach*: The first benchmark [69] jointly considers the selection of edge nodes and resource allocation to optimize energy consumption, delay, and computing capabilities. We employ this approach with the same parameter settings for fair comparison.
- *SAC-based approach*: The second benchmark is a traditional approach referred to as SACT, which sets resource allocation to cloudlets based on available computing capacity.
- *DDPG-based approach*: The third benchmark, DDPG, makes resource allocation decisions based on environmental feedback.
- *Structure2Vec approach*: The forth benchmark is Structure2Vec (referred to as S2Vec), which facilitates learning through feature-embedding strategies.
- *Random approach*: The fifth benchmark randomly chooses cloudlets for resource allocation.
- *Greedy approach*: The sixth benchmark selects cloudlets greedily based on resource availability, considering the available bandwidth in links by assessing the closest paths.

### 7.2. Performance Analysis

**Figure 3:**
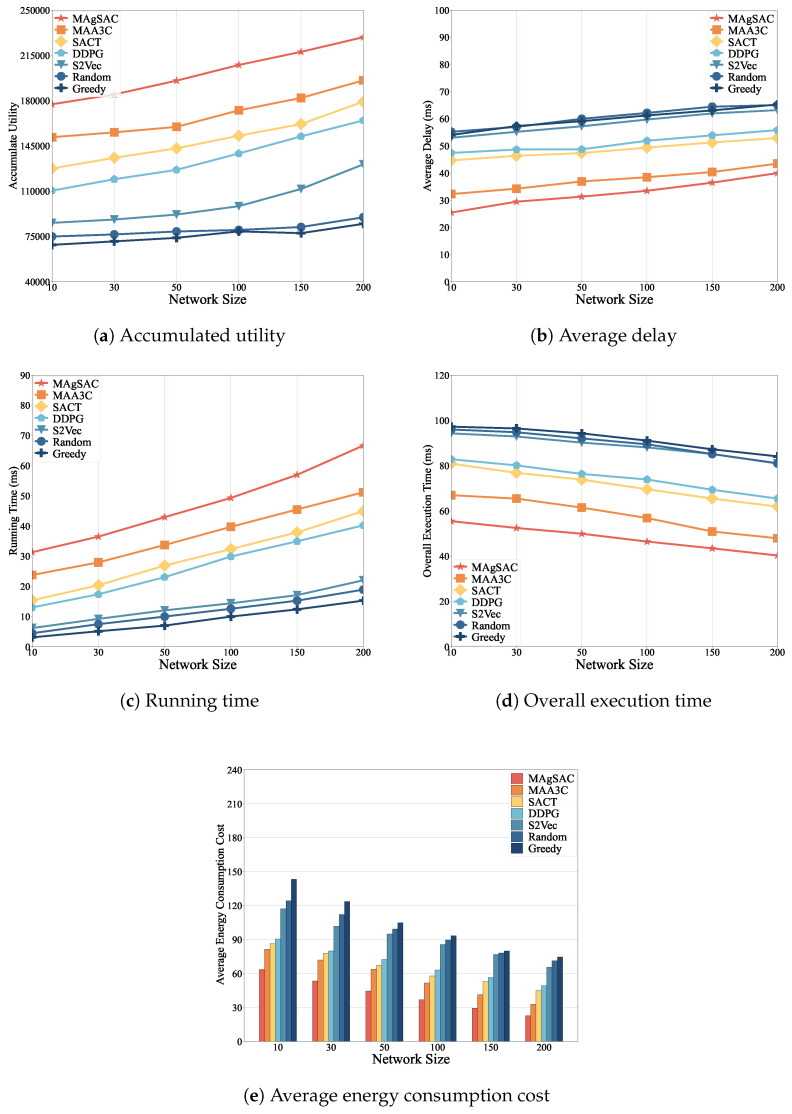
Performance comparison of the algorithms with respect to the network sizes varying from 10 to 200.

We first examine the performance of the MAgSAC algorithm against the other six benchmark algorithms (MAA3C, SACT, DDPG, S2Vec, Random, and Greedy) in terms of accumulated utility, end-to-end average delay, running time, overall execution time, and average energy consumption, with network sizes varying from 10 to 200 nodes, while setting the number of requests to 100. The proposed algorithm’s results are shown in Figure 3.

As we can see from Figure 3a, the MAgSAC algorithm has a higher accumulated utility than the benchmark algorithms, outperforming MAA3C by 19.9%, SACT by 35.2%, DDPG by 49.8%, S2Vec by 104.2%, Random by 147.2%, and Greedy by 167.7%. The reason is that when the network size is 10, MAgSAC has fewer activated VNF instances available, and with a network size of 200, not only are more VNF instances available to facilitate IoT user requests, but the algorithm also utilizes the remaining capacity and idle VNF instances efficiently. However, the Random and Greedy approaches gain the lowest utility compared to MAA3C, SACT, DDPG, and S2Vec because they choose to activate new VNF instances rather than utilize existing ones. When the network size is 100, the Random and Greedy algorithms perform almost identically due their choice of the closest path to accommodate user demand.

In Figure 3b, it can be observed that the end-to-end average delay experienced by MAgSAC is much lower than that of its counterpart algorithms, with an average delay 13.1% less than that of MAA3C, 32.7% less than that of SACT, 35.9% less than that of DDPG, 43.9% less than that of S2Vec, 46.2% less than that of Random, and 45.9% less than that of Greedy. The reason is that MAgSAC intelligently finds the activated VNF instance in the primary cloudlet with minimum delay, maximizing utility.

Figure 3c represents the running time of all the comparison algorithms, where we can see that the running time of all the algorithms gradually increases from a network size of 10 to 200. However, the running time of MAgSAC is 27.8% higher than that of MAA3C, 59.5% higher than that of SACT, 78.9% higher than that of DDPG, 250% higher than that of S2Vec, 312.4% higher than that of Random, and 435.6% higher than that of Greedy. Compared to the six benchmark algorithms, MAgSAC obtains the highest utility with minimum delay and more feasible solutions than the other algorithms.

From Figure 3d, it can be seen that the overall execution time to compute the IoT user requests of all the algorithms varies with network size from 10 to 200. Algorithm MAgSAC performs much better than the other six comparison counterparts. When the network size is 10, the execution time is 59.3, meaning most requests are implemented in the home cloudlet by assigning the activated idle or remaining capacity of VNF instances, and fewer requests are referred to the adjacent cloudlet. As the network size increases, more VNF instances are available to accommodate the requests, and the execution time decreases up to a network size of 200. On the other hand, when the network size is 150 and 200, the execution time of the S2Vec and Random algorithms is identical because the feature-embedding strategy of S2Vec and the random resource selection employed in the cloudlet of Random deliver similar results. Thus, the overall execution time to compute the requests of MAgSAC is 17.4% lower than, and 435.6% higher than that of MAA3C, 33% lower than, and 435.6% higher than that of SACT, 35.6% lower than, and 435.6% higher than that of DDPG, 45.8% lower than, and 435.6% higher than that of S2Vec, 47% lower than, and 435.6% higher than that of Random, and 48.6% lower than, and 435.6% higher than that of Greedy.

Figure 3e depicts the average energy consumption costs to implement IoT user requests to activate and deactivate the VNF instances of all the algorithms. The MAgSAC algorithm has the lowest cost to implement user requests compared to the other six algorithms, even when active resources are limited at a network size of 10. As the network size increases, the cost decreases. The reason is that with increasing network size, more VNF instances finish processing and become available for reuse, and our algorithm chooses the lowest-cost implementation resources with minimum delay, maximizing total utility. Thus, the average energy consumption cost to compute the requests using MAgSAC is 27% less than that using MAA3C, 35.2% less than that using SACT, 39.2% less than that using DDPG, 53.7% less than that using S2Vec, 56.3% lower than that using Random, and 59.5% lower than that using Greedy.

Next, we examine how the number of cloudlets impacts performance in a real network topology, namely AS1755, ranging from 10 to 100 cloudlets, using the MAgSAC, MAA3C, SACT, DDPG, S2Vec, Random, and Greedy algorithms.

**Figure 4:**
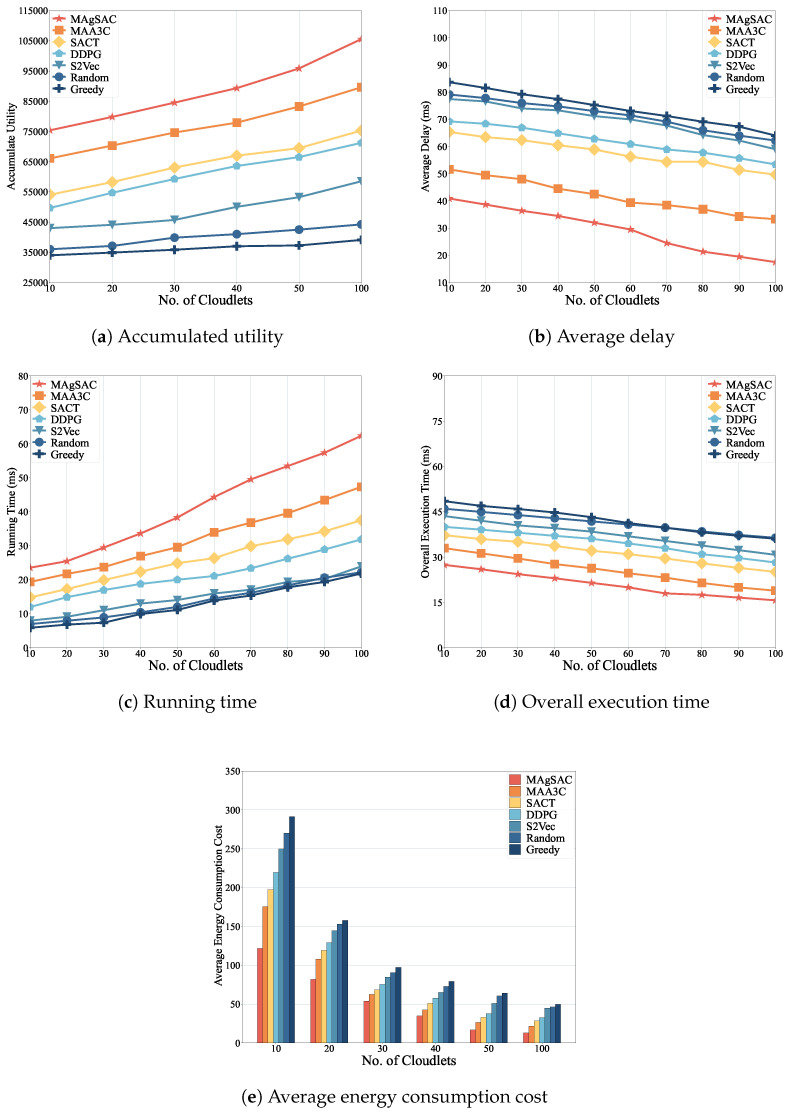
Performance comparison of the algorithms with respect to the No. of cloudlets in a real network (AS1755).

In Figure 4a, we can notice that the MAgSAC algorithm delivers the highest utility compared to the other six algorithms, corresponding to 18.1% more than MAA3C, 40.9% more than SACT, 49.4% more than DDPG, 85.2% more than S2Vec, 127.6% more than Random, and 150.3% more than Greedy, with Greedy having the lowest utility among all algorithms. The reason is that with the growth in the number of cloudlets, more user requests are implemented by considering the requirements and available resources. This also minimizes the end-to-end average delay, providing resources not only for common requests but also for urgent IoT user requests. The delay of the MAgSAC algorithm dramatically decreases by 31.3% compared to MAA3C, 48.9% compared to SACT, 53% compared to DDPG, 58.3% compared to S2Vec, 59.4% compared to Random, and 61% compared to the Greedy benchmark when the number of cloudlets ranges from 60 to 100, as we can observe in Figure 4b.

Despite this, a high number of cloudlets may result in a longer search for an optimum solution, thereby unavoidably prolonging the running time of all seven algorithms. The running time of MAgSAC is 29.3% more than that of MAA3C, 57.7% more than that of SACT, 102.7% more than that of DDPG, 166.4% more than that of S2Vec, 182% more than that of Random, and 252.8% more than that of the Greedy method, as shown in Figure 4c.

Figure 4d presents the overall execution times of the algorithms. The overall execution time of MAgSAC is 14% lower than that of MAA3C, 29.7% lower than that of SACT, 36.4% lower than that of DDPG, 42.4% lower than that of S2Vec, 46.6% lower than that of Random, and 47% lower than that of Greedy. Since MAgSAC enables the agents to learn and improve the policy, it efficiently mitigates the execution time with the increasing number of cloudlets, maximizing the accumulated utility and reducing the overall energy consumption cost to activate and deactivate the VNF instances according to the requirements of IoT users. This results in the lowest energy consumption cost of MAgSAC, which is 26.4% lower than that of MAA3C, 35.3% lower than that of SACT, 41.6% lower than that of DDPG, 49.5% lower than that of S2Vec, 53.5% lower than that of Random, and 56.4% lower than that of Greedy, as can be observed in Figure 4e.

**Figure 5:**
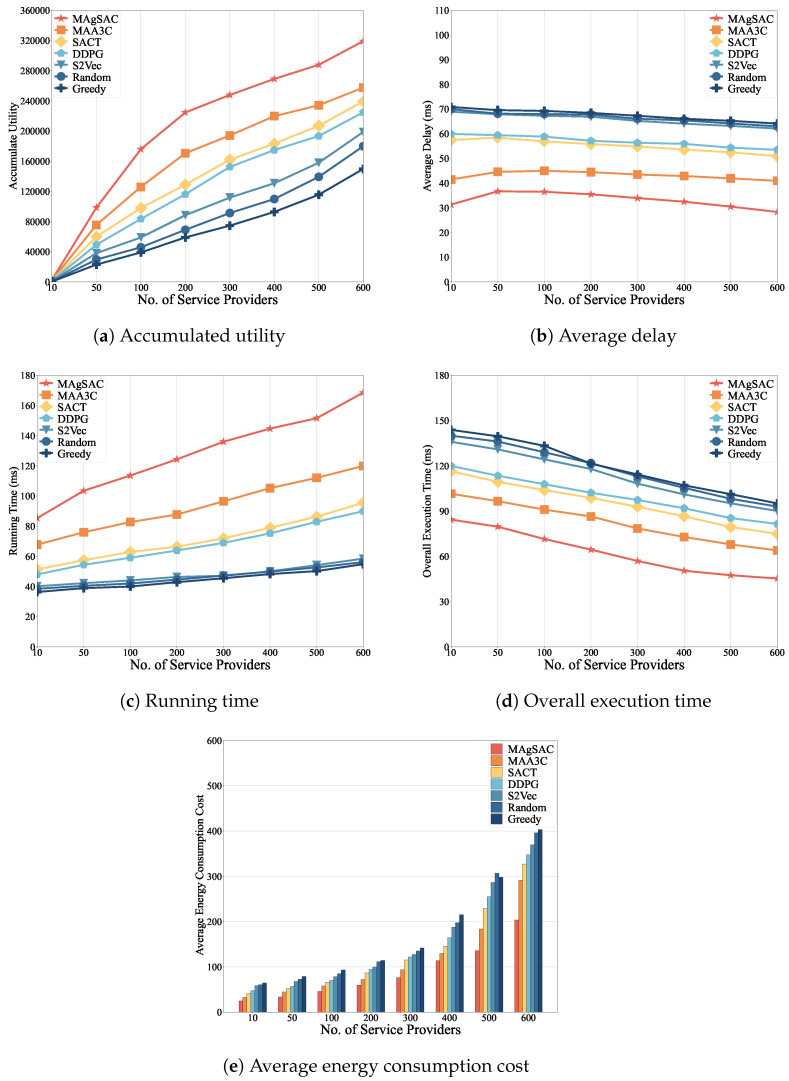
Performance comparison of the algorithms with respect to the No. of service providers in a real network (AS1755).

Next, we investigate the impact of the number of service providers on the performance of the MAgSAC, MAA3C, SACT, DDPG, S2Vec, Random, and Greedy algorithms by varying the number of service providers in a real network topology (AS1755). Results of the accumulated utility, average delay of IoT user requests, running time, overall execution time of the requests, and average energy consumption cost are defined in Figure 5. In Figure 5a, we can see that the accumulated utility gained by MAgSAC and MAA3C increases with the growth in the number of service providers compared to the SACT, DDPG, S2Vec, Random, and Greedy algorithms. The reason is that more resources are allocated by the service providers to the users, which gradually increases the accumulated utility. The utility achieved by MAgSAC is 30% higher than that of MAA3C, 44% higher than that of SACT, 57.9% higher than that of DDPG, 57.4% higher than that of S2Vec, 85% higher than that of Random, and 118% higher than that of the Greedy strategy. However, the end-to-end average delay of all seven compared counterparts slightly decreases. The average delay of MAgSAC is 22.4% lower than that of MAA3C, 43.7% lower than that of SACT, 45.4% lower than that of DDPG, 47.6% lower than that of S2Vec, 47.7% lower than that of Random, and 48.2% lower than that of Greedy, as seen in Figure 5b.

Meanwhile, as shown in Figure 5c, the running time of all the algorithms increases with the growth of service providers, but MAgSAC has the highest running time of almost 68.7 ms. The strategy of MAgSAC for allocating resources requires more time, including training time, to reach the optimal level, which is acceptable. The increasing running time percentage of MAgSAC is 37.4% more than that of MAA3C, 71% more than that of SACT, 89.3% more than that of DDPG, 161.3% more than that of S2Vec, 176.4% more than that of Random, and 205.7% more than that of Greedy.

Figure 5d illustrates the overall execution time of all seven algorithms. We can see that the execution time required by MAgSAC is 21.7% lower than that of MAA3C, 34.5% lower than that of SACT, 37.4% lower than that of DDPG, 44.7% lower than that of S2Vec, 46.6% lower than that of Random, and 47.6% lower than that of Greedy from 10 to 600 service providers. The reason is that an optimal number of shared resources from service providers is needed to execute IoT user requests. More resource availability not only minimizes the delay but also the execution time. We can also observe that the execution time of the Random and Greedy algorithms is almost identical with 200 to 400 service providers. This is because the Random algorithm chooses resources to fulfill the demand randomly, which is excessive with 200 service providers, and the Greedy algorithm allocates resources based on availability. Therefore, the two strategies perform identically in this range.

On the other hand, the average energy consumption cost obtained the MAgSAC algorithm is 12.4% lower than that of MAA3C, 26% lower than that of SACT, 31.4% lower than that of DDPG, 37.7% lower than that of S2Vec, 41.8% lower than that of Random, and 44% lower than that of Greedy with an increasing number of service providers, as evidenced in Figure 5e. The reason behind this decrease is that more service providers are involved in the process of resource allocation, which not only increases the utility of the service providers but also increases the processing cost of IoT user requests, including the bandwidth utilization cost in the links.

**Figure 6:**
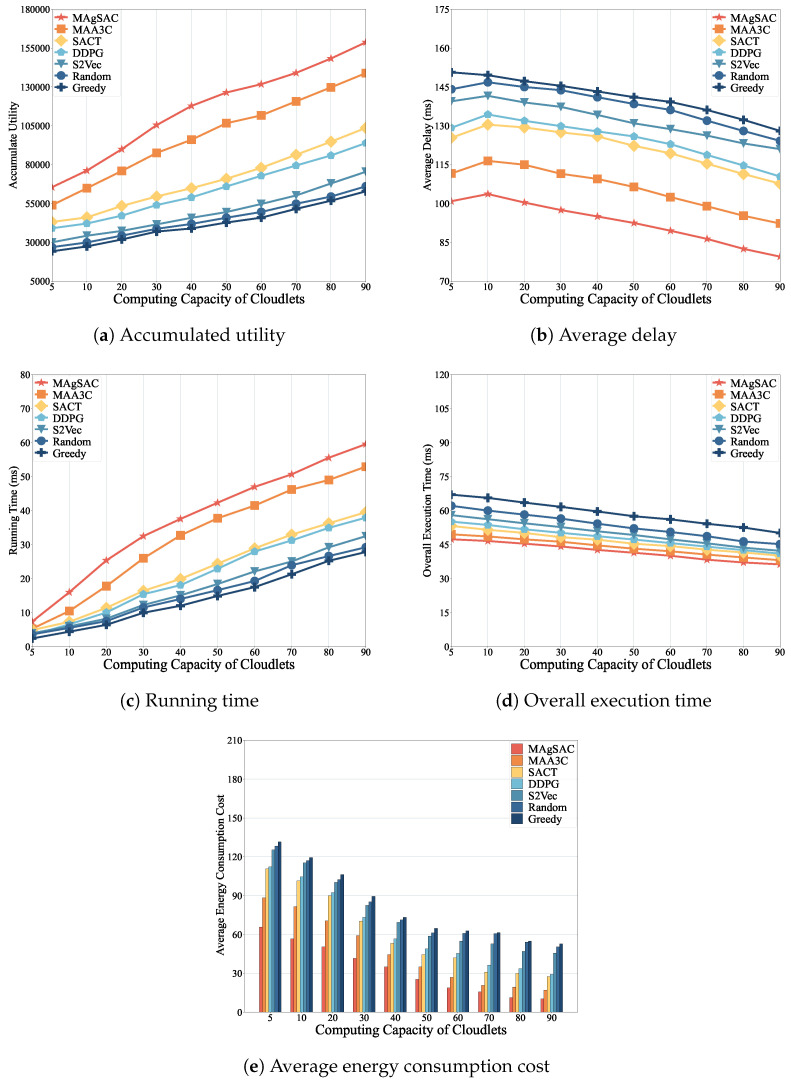
Performance comparison of the algorithms with respect to the computing capacity of the cloudlets in a real network (AS1755).

We now investigate the impact of the computing capacity of the cloudlets in a real network topology (AS1755) from 5 MHz to 90 MHz on the performance of the seven benchmark algorithms, namely MAgSAC, MAA3C, SACT, DDPG, S2Vec, Random, and Greedy, in terms of accumulated utility, average end-to-end delay, running time, overall execution time, and average energy consumption cost, as depicted in Figure 6. We can see that the MAgSAC algorithm gains around 8.7%, 37.5%, 54.1%, 93.7%, 114%, and 135.8% accumulated system utility relative to the MAA3C, SACT, DDPG, S2Vec, Random, and Greedy algorithms, then remains stable afterwards when the cloudlet computing capacity is 90 MHz. The reason is that the computing capacity of the cloudlets is stable, achieving more utility with the growth of the capacity, as shown in Figure 6a.

The end-to-end average delay of all the algorithms can be seen in Figure 6b. The delay from 5 MHz to 10 MHz is slightly higher for all the algorithms, although that of MAgSAC is 8% lower than that of MAA3C, 19.6% lower than that of SACT, 21.9% lower than that of DDPG, 27% lower than that of S2Vec, 30% lower than that of Random, and 30.8% lower than that of Greedy. As the computing capacity of the algorithms increases, the delay decreases because more capacity is available, and with minimal delay, more requests are implemented.

Figure 6c shows the running time of all the comparison algorithms. We can observe that the running time of MAgSAC and MAA3C is higher than that of the other algorithms due to the improved policy in the resource allocation process. However, the running time of the SACT, DDPG, S2Vec, Random, and Greedy algorithms shows a gradually reducing but steady trend with the growth of the computing capacity of the cloudlets. The running time of MAgSAC is 20.3% more than that of MAA3C, 53% more than that of SACT, 56.1% more than that of DDPG, 77.5% more than that of S2Vec, 80.4% more than that of Random, and 100.3% more than that of Greedy.

We further investigate the impact of increasing the computing capacity of the cloudlets on all the algorithms, as seen in Figure 6d. The findings demonstrate that when the computing capacity of the cloudlets improves, the overall time taken to implement the requests decreases. Each scheme exhibits a consistent pattern as a consequence of the enhanced computational capabilities of MEC networks. As the computing capacity of the cloudlets grows, our MAgSAC approach demonstrates a significant reduction in overall execution time compared to the other six algorithms, with an overall execution time 0.27% lower than that of MAA3C, 1.2% lower than that of SACT, 2.2% lower than that of DDPG, 6.5% lower than that of S2Vec, 11.3% lower than that of Random, and 18.8% lower than that of Greedy.

The average energy consumption costs of the MAgSAC, MAA3C, SACT, DDPG, S2Vec, Random, and Greedy algorithms are presented in Figure 6e. The average energy consumption cost computing IoT user requests decreases with the growth of the computing capacity of all seven algorithms. This is because with the increase in computing capacity of the cloudlets, more users are accommodated by the service providers with the available resources without activating new VNF instances, which gradually decreases the cost with increasing cloudlet capacity. Therefore, in terms of average energy consumption cost regarding computing capacity of the cloudlets, MAgSAC achieves the lowest cost that is 17.6% lower than that of MAA3C, 29.3% lower than that of SACT, 30.6% lower than that of DDPG, 41.7% lower than that of S2Vec, 43.2% lower than that of Random, and 44.5% lower than that of Greedy.

**Figure 7:**
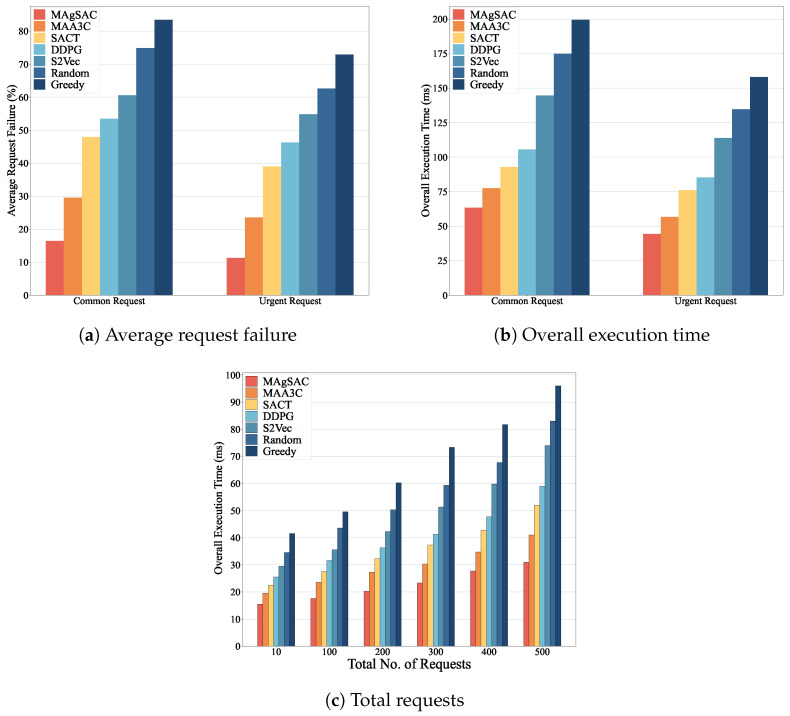
Performance comparison of the algorithms with respect to the requests in a real network (AS1755).

Figure 7 shows the impact of the number of requests on the performance of the MAgSAC, MAA3C, SACT, DDPG, S2Vec, Random, and Greedy algorithms. The primary purpose of our approach is to facilitate both common and urgent IoT user requests as much as possible to minimize the failure percentage within the given time frame. Minimizing request failure is very important in emergency cases where an autonomous vehicle or real-time application needs a quick response. The common-request failure percentages of the seven comparison algorithms are slightly higher than those of the urgent-request algorithms, as seen in Figure 7a. However, our algorithm (MAgSAC) has a request failure percentage that is 44.3% lower than that of MAA3C, 65.7% lower than than that of SACT, 69.2% lower than than that of DDPG, 72.8% lower than than that of S2Vec, 78% lower than than that of Random, and 80% lower than than that of Greedy for common requests. For urgent requests, it is 52% lower than than that of MAA3C, 71% lower than than that of SACT, 75.5% lower than than that of DDPG, 79.3% lower than than that of S2Vec, 81.9% lower than than that of Random, and 84.5% lower than than that of Greedy.

The higher percentage of common-request failures is due to our prioritization of urgent requests. In primary cloudlets, all urgent requests are handled with minimal execution time, which results in common requests being transferred to adjacent cloudlets when resources are unavailable in the home cloudlet. This prioritization is reflected in the higher execution time for common requests, as evidenced in Figure 7b. The overall request execution time for MAgSAC is 18.1% less than that of MAA3C, 31.7% less than that of SACT, 39.8% less than that of DDPG, 56.1% less than that of S2Vec, 63.7% less than that of Random, and 68.1% less than that of Greedy for common requests. For urgent requests, the execution time of MAgSAC is 21.6% less than that of MAA3C, 41.7% less than that of SACT, 48% less than that of DDPG, 61% less than that of S2Vec, 67% less than that of Random, and 71.9% less than that of Greedy.

Figure 7c shows that the overall execution time of the requests increases with the growth of the total number of requests. The rationale behind this is that as the number of requests increases, not only does prediction require differentiation common and urgent requests, but appropriate resources must also be allocated according to the requirements. In some cases, requests need to be transferred to adjacent cloudlets for implementation, and congestion may occur, which increases the overall execution time. The execution time for the MAgSAC algorithm is 23.2% lower than that of MAA3C, 36.8% lower than that of SACT, 45.9% lower than that of DDPG, 53.6% lower than that of S2Vec, 59.9% lower than that of Random, and 66.3% lower than that of Greedy. Thus, the MAgSAC algorithm has the lowest execution time among all the comparison counterparts.

**Figure 8:**
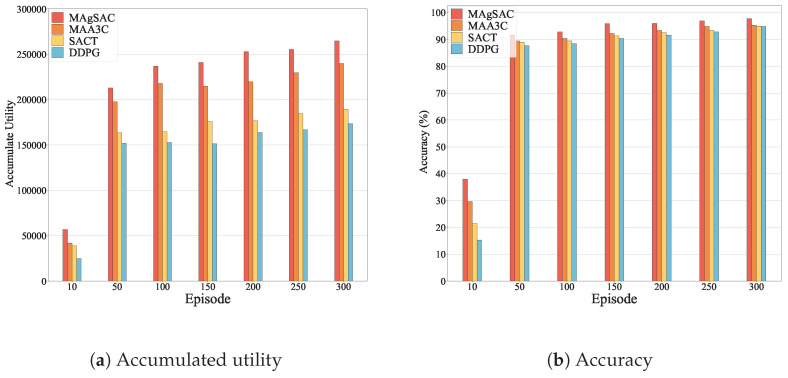
Comparison of the convergence performance of the algorithms with respect to the number of episodes.

Finally, we evaluate the convergence performance of our proposed MAgSAC approach in comparison with three DRL-based benchmarks, namely MAA3C, SACT, and DDPG, based on MINIST [70], as shown in Figure 8. In Figure 8a, we can notice that the accumulated utility over the first 10 episodes is at the lowest level but gradually increases and starts converging to an approximate optimal point from episode 50 onwards. The MAgSAC, MAA3C, SACT, and DDPG algorithms all converge to a stable solution from episode 50 onwards, which means that as the number of episodes grows, the speed of convergence tends to increase for all the algorithms. While the SACT and DDPG algorithms aim to maximize the estimated long-term return, their overall utility is slightly lower than that obtained by the other two algorithms, namely MAgSAC and MAA3C. The notable enhancement of the proposed MAgSAC approach and the MAA3C scheme may be due to the use of a maximum-entropy regularized stochastic strategy instead of a deterministic policy. Furthermore, our proposed MAgSAC algorithm clearly achieves more rapid convergence and superior utility, outperforming MAA3C by 21.8%, SACT by 38.7%, and DDPG by 51.8%. The proposed MAgSAC algorithm achieves convergence after around 50 episodes. The reason is that the proposed MAgSAC method allows for the efficient allocation of resources not only in the home cloudlet but also in adjacent cloudlets, thereby preventing the system from reaching a sub-optimal solution.

In Figure 8b, we examine the accuracy achieved by our proposed MAgSAC algorithm against the other DRL-based algorithms, namely MAA3C, SACT, and DDPG. It is worth mentioning that all algorithms indicate an immediate rise in accuracy during the first 10 episodes, then gradually reach a stable state after about 50 rounds. Efficiency reduction may occur only when there is a lack of resources in the cloudlets of concern. The results indicate that MAgSAC, MAA3C, SACT, and DDPG exhibit superior accuracy. The accuracy gain by MAgSAC is 38% at 10 episodes, which is much higher than that of the comparison counterparts. Afterwards, with the increase in episodes, the accuracy percentage increases to 4.2% higher than that of MAA3C, 6.4% higher than that of SACT, and 8.7% higher than that of DDPG. However, MAgSAC outperforms all of them, obtaining an accuracy rate of over 97% in up to 300 episodes. It is evident that as the number of episodes increases, our technique improves significantly. Therefore, the MAgSAC algorithm achieves exceptional performance, clearly showing its superiority compared to previously reported techniques.

## 8. Research Findings

Our work presents a MAgSAC algorithm designed for efficient network slicing in MEC environments. This research addresses the challenge of uneven traffic distribution and resource constraints by predicting and classifying IoT requests as common or urgent, thereby optimizing resource allocation and minimizing latency. The proposed MAgSAC algorithm enhances the management of VNF instances within cloudlets, preventing overloading and ensuring balanced resource distribution. By transforming the optimization problem into an MDP, the algorithm tackles the complexity of resource allocation through intelligent decision making. Extensive simulations demonstrate that the MAgSAC approach outperforms six benchmark methods, namely MAA3C, SACT, DDPG, S2Vec, Random, and Greedy, in terms of accumulated utility, energy consumption, and execution time. It successfully balances trade-offs between revenue, energy costs, and latency while ensuring the timely completion of both common and urgent requests. Additionally, the algorithm addresses the challenge of adapting to real-time changes in user demand and resource availability. These findings highlight the algorithm’s effectiveness in optimizing MEC systems, ensuring high QoS for diverse IoT applications, and mitigating potential risks associated with resource limitations.

## 9. Conclusions

This paper addresses the challenge of resource allocation in MEC environments, particularly the uneven distribution of IoT requests. We propose a network slicing-based MEC system that utilizes a MAgSAC algorithm. Our approach leverages strategically placed cloudlets with on-demand VNF activation/deactivation capabilities to efficiently cater to both common and urgent IoT requests. To optimize the resource allocation process, we formulate a comprehensive optimization model that considers utility, energy consumption cost, and latency. By transforming this model into a multi-agent deep reinforcement learning problem, we achieve intelligent resource allocation through the use of the MAgSAC algorithm while optimizing overall utility. Extensive simulations demonstrate the effectiveness of our approach, achieving the highest utility, minimizing execution time, and reducing energy consumption costs compared to existing methods. Our work paves the way for efficient resource allocation in MEC systems, ensuring optimal QoS for diverse IoT users.

Future research can build on this foundation by investigating new optimization parameters, extending the system’s scalability, and incorporating more advanced prediction models to further optimize resource allocation techniques in MEC scenarios. Furthermore, investigating the integration of serverless computing paradigms could lead to novel solutions to address complex resource allocation challenges by disassociating resource management from individual instances. Additionally, evaluating the impact of practical constraints, such as network latency and device heterogeneity, on the proposed method’s efficiency would be advantageous. Future research could explore the enhancement of the MAgSAC algorithm’s adaptability to dynamic and unpredictable IoT traffic patterns. Comparative analysis with other cutting-edge DRL techniques could offer a deeper comprehension of the algorithm’s performance and potential areas for enhancement.

## Figures and Tables

**Table 1:** Terminology.

Term	Definition
$G=\left(Z\cup V,E\right)$	MEC network, where Z is a set of cloudlets, V is a set of APs, and E is a set of edge links
$F_{l}$	A set of VNF instances
$z_{j}^{cap}$	Capacity of a cloudlet $z_{j}$
$f_{l}^{⊕}$	An activated VNF instance
$f_{l}^{⊘}$	A deactivated VNF instance
$p(f_{l})$	Computing resource requirement of VNF instance $f_{l}$
*t*	Time-slot index 1≤t≤T
$B_{e}$	Bandwidth at the edge (*e*)
$D_{e}$	Delay at the edge (*e*)
E(i,j)	A set of links
$r_{i}^{s}\in R^{s}$	Network slice requests
λ	Arrival rate of requests
$\varrho_{j,l}^{t}$	Total number of unfinished requests
$\rho_{i,j}^{t}$	Request currently being processed
$r_{i}^{s}$	Common requests
$r_{urg}^{s}$	Urgent request
$SD_{i}$	T distribution
Δ	Standard deviation
$U^{up}$	Upper bound
$U^{lo}$	Lower bound
η	Average T distribution
$d_{n}^{u,h}$	Overall computational delay in the home cloudlet for urgent requests
$d_{qw^{\prime }}^{r^{s}}$	Additional queue delay in adjacent cloudlet
$d_{\bar{qw}}^{r^{s}}$	Queue waiting delay
$d_{n}^{u,ad}$	Overall computational delay ub adjacent cloudlet for urgent requests
$\chi_{n}^{r^{s,t}}$	A binary decision variable {0,1}
$d_{n}^{c,h}$	Overall computational delay in home cloudlet for common requests
$d_{n}^{c,ad}$	Overall computational in adjacent cloudlet for common requests
$b_{k}^{t}$	Required bandwidth to transfer a request via the $k^{th}$ link
$D_{j}^{t}$	Overall delay faced by common and urgent requests in the home and adjacent cloudlet
$c(f_{l})$	Computing cost of accommodating one unit of traffic
$c_{n}^{u,h}$	Overall energy consumption cost for urgent requests in the home cloudlet
$c_{n}^{u,ad}$	Overall energy consumption cost for urgent requests in the adjacent cloudlet
$c_{n}^{c,h}$	Overall energy consumption cost for common requests in the home cloudlet
$c_{n}^{c,ad}$	Overall energy consumption cost for common requests in the adjacent cloudlet
$C_{j}^{t}$	Overall energy consumption cost for common and urgent requests in the home and adjacent cloudlet
$PR_{j}^{t}$	Total profit earned by the cloudlet
${\left(f_{l}^{⊕}\right)}^{idl}$	An idle VNF instance
$z_{j}^{*cap}$	Remaining computing capacity of a cloudlet $z_{j}$
$\phi_{j,l}^{t}$	The decision on the assignment of resources for action $a_{i}^{t}$
$\theta_{i}^{\iota }$	Critic network with parameters $\theta_{i}^{\iota }$ (ι=1,2)
${\tilde{\theta }}_{i}^{\iota }$	Target network
$\alpha_{i}$	Temperature parameter
$B_{r}$	Experience reply buffer
$B_{\varpi }$	Mini-batch
*£*	Loss function
$\pi_{\zeta_{i}}$	Policy parameter
$a^{t}$	Action space
$s^{t}$	State space

## Data Availability

The data used in this study are included within the article.
